# OptoChaperone—A Biohybrid Tool for Regulating Protein Condensates in Cells and In Vitro

**DOI:** 10.1021/jacs.6c04074

**Published:** 2026-04-20

**Authors:** Do Thanh Tuan, Motonori Matsusaki, Honoka Ota, Soichiro Kawagoe, Hettimudalige Dilini Nisansala, Munehiro Kumashiro, Noriyoshi Isozumi, Hiroyuki Kumeta, Yohei Kono, Takeshi Shimi, Koichiro Ishimori, Eiichiro Mori, Satoshi Arai, Tomohide Saio

**Affiliations:** † Department of Physiology, 106156 Hanoi Medical University, Hanoi 100000, Vietnam; ‡ Graduate School of Medicine, 13109 Tokushima University, Tokushima, Tokushima 770-8503, Japan; § Institute of Advanced Medical Sciences, Tokushima University, Tokushima, Tokushima 770-8503, Japan; ∥ Institute of Photonics and Human Health Frontier, Tokushima University, Tokushima, Tokushima 770-8501, Japan; ⊥ Graduate School of Chemical Sciences and Engineering, Hokkaido University, Sapporo, Hokkaido 060-8628, Japan; # Graduate School of Frontier Science Initiative, Division of Nano Life Science, Kanazawa University, Kakuma-machi, Kanazawa 920-1192, Japan; ¶ Department of Future Basic Medicine, 12967 Nara Medical University, Kashihara, Nara 634-8521, Japan; ∇ Faculty of Advanced Life Science, Hokkaido University, Sapporo, Hokkaido 001-0021, Japan; ○ Nano Life Science Institute (WPI-NanoLSI), Kanazawa University, Kakuma-machi, Kanazawa 920-1192, Japan; ⧫ Department of Chemistry, Faculty of Science, Hokkaido University, Sapporo, Hokkaido 060-0810, Japan

## Abstract

Protein condensates formed via liquid–liquid phase separation (LLPS) are increasingly recognized as key players in diverse cellular processes, including those associated with disease. Despite extensive efforts to characterize their formation and function, tools that enable precise, reversible, and spatiotemporal control of LLPS remain limited. Here, we report OptoChaperone, a light-activatable molecular system designed to manipulate protein condensates both in vitro and in living cells. This biohybrid system leverages photoresponsive switching to control chaperone activity: blue light triggers the suppressive function, leading to the dissolution of protein condensates, whereas UV light deactivates the system, allowing condensate formation. We demonstrate the efficacy of OptoChaperone in regulating several disease-related protein condensates, such as fused in sarcoma, TAR DNA-binding protein 43, and heat shock factor 1. Importantly, the system exhibits reversible and robust control over droplet dynamics without requiring chemical additives or genetic modifications of the client proteins. Given the reversibility and efficiency of OptoChaperone in the manipulation of protein condensates, this tool offers a powerful platform for dissecting the roles of protein condensation in cellular physiology and pathology. This strategy also holds potential for broader applications in synthetic biology, biomolecular engineering, and therapeutic modulation of aberrant phase separation.

## Graphical abstract

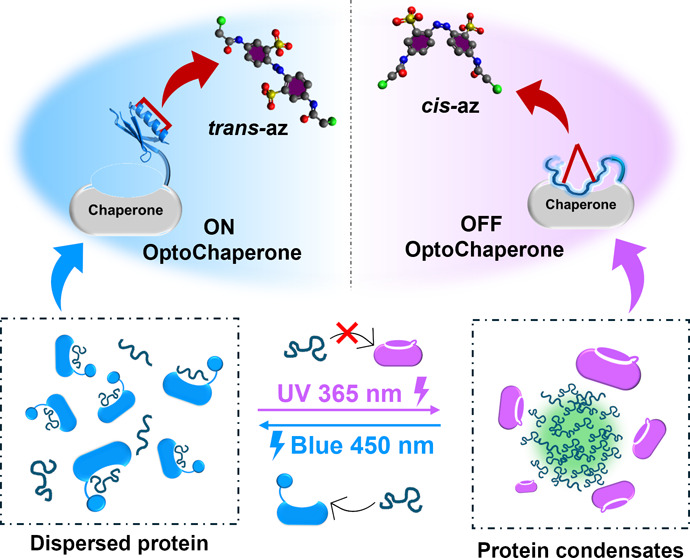

## Introduction

Protein aggregation is a hallmark of various neurodegenerative diseases,^1–3^ including amyotrophic lateral sclerosis,^4^ frontotemporal dementia,^5^ Parkinson’s disease,^6^ and Alzheimer’s disease.^7,8^ Many disease-associated proteins, such as fused in sarcoma (FUS),^9^ TAR DNA-binding protein 43 (TDP-43),^10^ α-synuclein,^11,12^ and tau^13^ undergo condensation—most commonly through liquid–liquid phase separation (LLPS)—as a prerequisite for pathological aggregation.^14–16^ Protein condensation is also closely related to protein function as seen in stress response,^17^ transcription regulation, RNA splicing,^18^ and signal transduction,^19^ where protein condensation assembles multiple components and increases the local concentration of the selected molecules. However, the functional significance of protein condensation remains elusive, in part due to the lack of technologies for spatiotemporal and reversible control over protein condensates.

Several physical and chemical parameters, including pH, temperature, redox state, and salt concentration, can influence protein condensates.^20–22^ However, these global environmental changes affect not only protein condensation but also many other cellular events. Optogenetic systems have offered a significant advancement in this field.^23^ Several optogenetic systems have been developed to control protein condensates in cells, including optoDroplet,^24^ PixELLs,^25^ Corelet,^26^ CasDrop,^27^ iPOLYMER-L,^28^ LOVTRAP.^29^ These systems typically function by fusing photoinducible oligomeric proteins with LLPS-potent proteins to enable light-induced droplet formation. While these systems have significantly advanced LLPS research, they are largely tailored for promoting condensate formation, and often lack reversibility, leading to persistent or irreversible assemblies. To address this limitation, OptoMBP was recently introduced as a tool for on-demand dissolution of condensates.^30^ However, because this recruitment-based strategy relies on scaffold-specific modifications, its versatility remains limited when targeting a broad range of different protein condensates without extensive re-engineering.

Here, we report OptoChaperone—a biohybrid optogenetic system that enables reversible regulation of protein condensates via a light-switchable molecular chaperone. Molecular chaperones have emerged as key factors in protein disassembly. For example, Hsp70 and Hsp27 are responsible for regulating TDP-43 and FUS condensates.^31,32^ Karyopherin-β2, also known as a nuclear transport receptor, dissolves FUS condensates.^33^ In this study, we designed OptoChaperone, utilizing a well-studied molecular chaperone trigger factor (TF) that recognizes unfolded proteins and intrinsically disordered proteins, including α-synuclein, to prevent the client proteins from self-assembling.^34–36^ In OptoChaperone, the chaperone activity is regulated by “lid protein” functionalized with an azobenzene derivative.^37–39^ Light-induced *trans*/*cis* isomerization of the azobenzene attached to the lid protein induces folding/unfolding of the lid protein, thereby modulating access to the client-binding site of the chaperone. We demonstrate that OptoChaperone enables precise, reversible control over protein condensates both in vitro and in cells, as shown using several disease-related protein condensates, including FUS,^40^ TDP-43,^41,42^ and heat shock factor 1 (HSF1).^17,43–45^ This light-responsive chaperone system provides a robust and generalizable strategy for dissecting LLPS-mediated mechanisms in cellular function and disease and opens new avenues for synthetic modulation of biomolecular condensates.

## Results

### Design of OptoChaperone

OptoChaperone was constructed by fusing TF chaperone with the immunoglobulin-binding domain B1 of a streptococcal protein G (GB1) mutant labeled with a photoswitchable compound azobenzene derivative 2,2′-bis­(sulfonato)-4,4′-bis­(chloroacetamido)­azobenzene (BSBCA),^37,46–48^ as a lid protein (Figure 1a,b). To immobilize BSBCA, the GB1 mutant was designed to have two exposed Cys residues (25 and 36) on the helix. The Cys residues were introduced at residue numbers *i* and *i* + 11, whose distance matches that of both ends of the BSBCA arms in the *trans* population.^37,46^ In contrast, the *cis* population of BSBCA has a reduced arm distance,^49^ leading to breakage of the helix.^37,50^ Thus, the GB1 mutant labeled with BSBCA, azGB1, is expected to unfold with 365 nm near-UV light, inducing the *cis* population of azobenzene. Given that TF binds unfolded proteins but not folded proteins,^34^ it is expected to selectively bind *cis*-azGB1, resulting in buried client binding sites on TF. With *trans*-azGB1, induced by 450 nm blue light, the client-binding sites on the TF are expected to be exposed, creating an active state that binds client proteins and suppresses condensation. Accordingly, azGB1was used as a “lid” and fused to the TF domains: the TF^PPD‑SBD^ consisting of peptidyl–prolyl isomerization domain (PPD: amino acid residues 150–246) and the substrate-binding domain (SBD: amino acid residues 113–149 and 247–432). Using the above design, the fusion protein azGB1-(Gly-Ser-linker)-TF^PPD‑SBD^, hereafter referred to as OptoChaperone, was designed (Figure 1c). The details of the design and preparation are described in the Methods Section.

**Figure 1:**
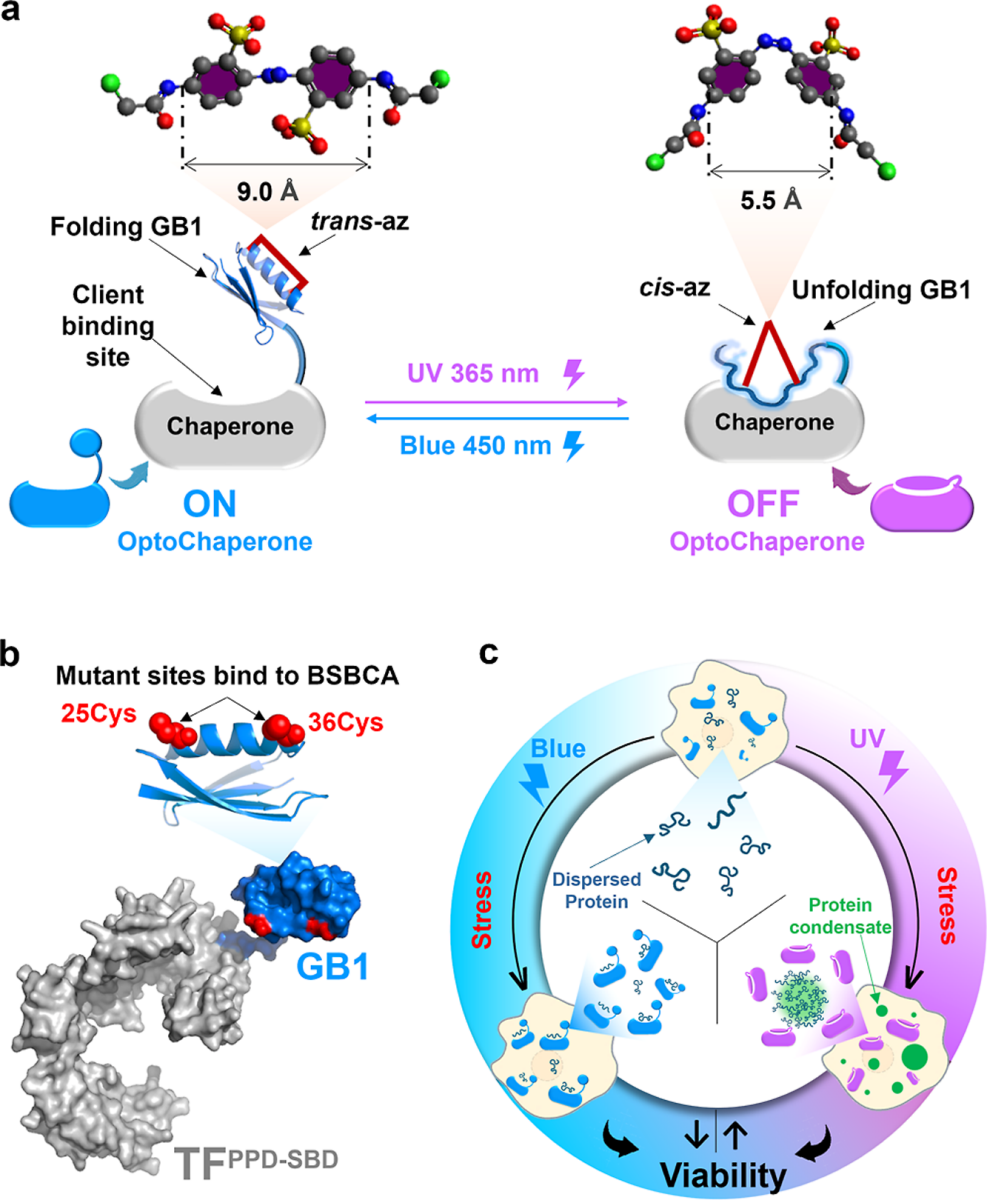
Schematic diagram of the new protein condensate light manipulation tool. (a) Structure of ON/OFF OptoChaperone and isomerization between different states by light irradiation. (b) Blueprint of a GB1-TF^PPD‑SBD^ protein containing mutations for BSBCA introduction. Domain arrangement and structure of a full-length OptoChaperone include GB1-solubilization tag with two Cys residue introduction for BSBCA modification; (GS)_5_-linker sequence; two domains of the trigger factor chaperone, peptidyl–prolyl *cis/trans* isomerase domain (PPD) residues numbered 150–246, and substrate-binding domain (SBD) residues numbered 113–149 and 247–432. (c) Conceptual model: OptoChaperone allows the reversible, light-dependent regulation of stress-responsive protein condensates in living cells, leading to modulation of stress response pathways and cell survival.

### Photoresponse of OptoChaperone

The conformational state of the BSBCA moiety in the lid azGB1 after light irradiation was evaluated using UV–vis spectroscopy using the formula described in the Methods Section (Figure 2a,b). The data showed that UV light increased *cis* conformation to 60%, whereas blue light induced *trans* conformation to 75% (Figure 2b). These results indicated that the *cis*/*trans* populations of BSBCA could be induced using UV/blue light irradiation. Notably, the *cis*/*trans* population changes were repeatable, with no significant deterioration. The structure of the GB1 moiety of azGB1 after light irradiation was evaluated using nuclear magnetic resonance (NMR). The ^1^H–^15^N heteronuclear single quantum coherence (HSQC) spectra of ^15^N-labeled GB1 mutant attached to BSBCA (^15^N-azGB1) after blue light irradiation showed well-dispersed resonances, indicating that GB1 has a tertiary structure (Figure 2c). In contrast, the spectra after UV light irradiation showed reduced intensities, especially for the dispersed resonances, whereas the increased intensities for the resonances were located at approximately 8 ppm in the proton dimension. As the intense resonances with this narrow dispersion in the proton dimension are characteristic of unfolded proteins, the NMR data suggested that blue and UV lights induced the folded and unfolded states of azGB1, respectively. Thus, we concluded that light-induced *cis*/*trans* isomerization of BSBCA successfully modified the structure of GB1 attached to BSBCA.

**Figure 2:**
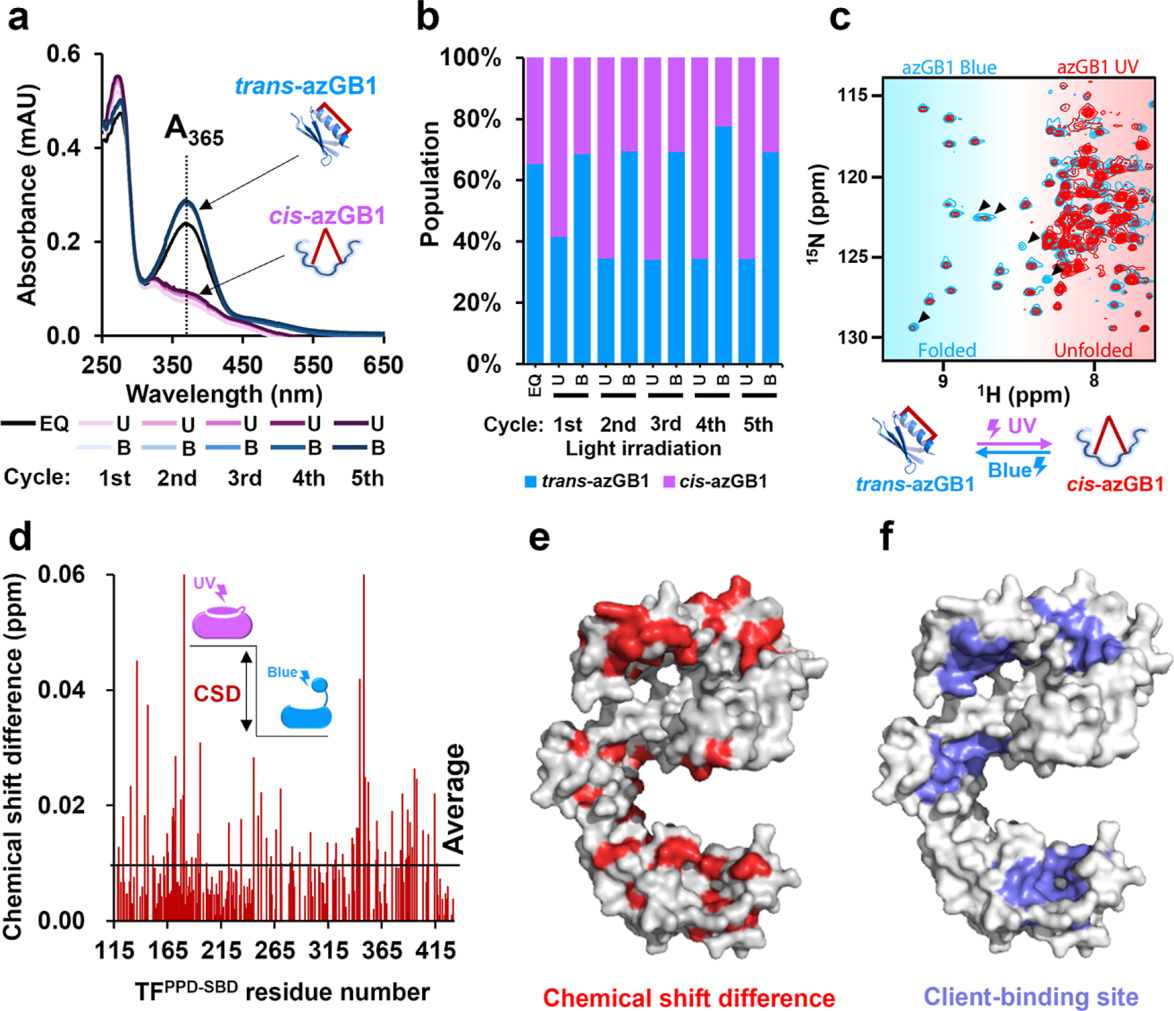
Investigation of properties of lid protein and OptoChaperone. (a) Ultraviolet–visible (UV–vis) spectra of azGB1 measured after alternate irradiation using blue light and UV light for 5 min. (b) Comparison between the ratios of *trans*-azGB1 and *cis*-azGB1 after exposure to light. (c) Superposition of the [^1^H–^15^N] HSQC spectra of *trans*-azGB1 (blue) and *cis*-azGB1 (red). The blue and red spectra were acquired after blue and UV light irradiation, respectively. The arrowheads represent GB1-derived signals where significant changes in signal intensity were observed. (d) Chemical shift difference (CSD) of amide moieties between *cis*-azGB1-TF^PPD‑SBD^ and *trans*-azGB1-TF^PPD‑SBD^, derived from the spectra in Figure S3a. The horizontal axis represents amino acid residues in OptoChaperone, and the vertical axis indicates the CSD. (e) CSD mapping (red) in OptoChaperone between *cis*-azGB1-TF^PPD‑SBD^ and *trans*-azGB1-TF^PPD‑SBD^. The residues showing the chemical shift perturbation larger than 0.01 ppm were mapped on the structure of TF^PPD‑SBD^ (PDB ID: 1w26). (f) Mapping of the client-binding sites on the surface area (blue) of TF^PPD‑SBD^ as reported previously.^34^ (EQ: thermal equilibrium, U: UV light irradiation, B: blue light irradiation).

Light-induced conformational changes were also evaluated based on UV–vis spectra. The UV–vis spectra after light irradiation showed that blue and UV light induced *trans*- and *cis*-OptoChaperone, respectively (Figure S1a–c). The conformational changes in GB1 fused within OptoChaperone were evaluated using NMR. ^1^H–^15^N transverse relaxation-optimized spectroscopy (TROSY) and ^1^H–^13^C heteronuclear multiple-quantum coherence (HMQC) spectra of OptoChaperone labeled with methyl-bearing (Ala, Ile, Leu, Met, and Val) residues were acquired after blue and UV light irradiation (Figures S2–S7). The resonances from the GB1 moiety appeared in the dispersed regions of the spectrum, with chemical shifts marginally corresponding to those of isolated GB1wt, showing that the GB1 moiety in the OptoChaperone holds its native fold (Figures S2a and S6). In contrast, the spectrum after UV irradiation showed a significant intensity reduction of the resonances from the folded GB1 moiety, indicating disruption of the GB1 structure, as seen for isolated azGB1 (Figures 2c and S2a,b). In the HSQC of azGB1, the folded components remained even after UV light irradiation (Figure 2c), whereas in the methyl HMQC spectra of OptoChaperone, the folded components were hardly visible. This may be because TF^PPD-SBD^ captures unfolded azGB1 and promotes its unfolding. . These data led us to conclude that the folded/unfolded state of the GB1 moiety in OptoChaperone could be reversibly switched using blue/UV light.

NMR data also showed a photoswitchable interaction between azGB1 and TF^PPD–SBD^ (Figures 2d and S3a). Chemical shift differences were evaluated for resonances from TF^PPD‑SBD^. Several specific residues, especially those from the client-binding sites,^34,35^ showed perturbations between the *trans* and *cis* states (Figure 2e,f). Additionally, the TF resonances from *cis*-OptoChaperone showed more significant perturbations from isolated TF^PPD‑SBD^ compared to those from *trans*-OptoChaperone (Figures S4 and S5), indicating that *cis*-Optochaperone has a higher population of GB1 moieties bound to TF^PPD‑SBD^ than *trans*-Optochaperone. These data suggest that photoswitching of the GB1 structure by BSBCA controls the interaction between GB1 and TF^PPD‑SBD^, highlighting the ability of azGB1 to function as a lid for TF chaperone. Regarding chaperone activity, *trans*-OptoChaperone should serve as an ON state or active state, while *cis*-OptoChaperone should serve as an OFF state or inactive state.

To guarantee the stability of ON and OFF OptoChaperone over prolonged periods in subsequent experiments in vitro and in cells, we investigated the lifetime of each state using UV–vis spectroscopy. The proteins were placed in the dark and incubated for up to 24 h, followed by UV–vis measurements. The data showed that the ON state was highly stable within 24 h, with a negligible change in absorbance over time (Figure S1d–f). Although the OFF state was less stable, it exhibited reasonable stability within 4 h. These data highlight sufficient stability for both the ON and OFF states of OptoChaperone for in vitro and in-cell regulation of protein condensates.

### OptoChaperone Regulates Protein Condensates In Vitro

The role of OptoChaperone in regulating protein condensates was evaluated in vitro. We first attempted to demonstrate protein condensate regulation by microscopic observation of HSF1 protein condensate induced in the presence or absence of the ON/OFF state OptoChaperone or unmodified chaperone, TF^PPD‑SBD^ (Figure 3a). With ON-state OptoChaperone, droplet formation was suppressed, and only small droplets were observed even after 60 min, indicating that OptoChaperone successfully suppressed HSF1 assembly (Figures 3a and S7). In contrast, OFF-OptoChaperone exhibited negligible suppression and allowed droplet formation. This result strongly supports the design of the OptoChaperone: UV light induces OFF-state OptoChaperone, losing its ability to bind HSF1 and triggering HSF1 condensation. Conversely, blue light induces ON state OptoChaperone, which captures HSF1 and prevents HSF1 self-assembly. We further corroborated the proposed mechanism by a fluorescence anisotropy assay using CF568-labeled HSF1 (Figure S8). The ON-state OptoChaperone induced increased fluorescence anisotropy, whereas the OFF-state OptoChaperone (UV light) induced negligible changes (Figure S8), supporting that the client protein is recognized selectively by ON-state OptoChaperone.

**Figure 3:**
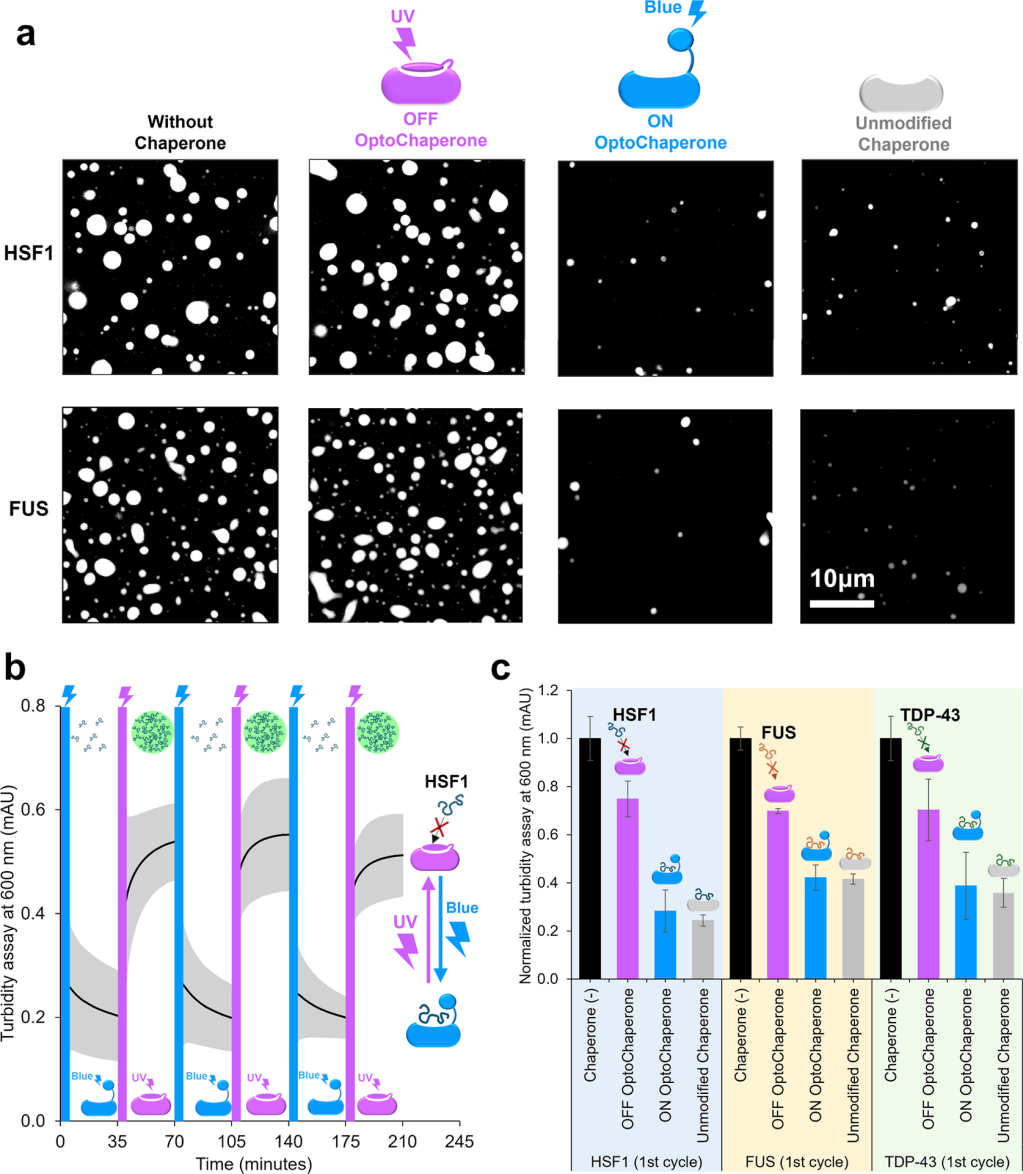
OptoChaperone enables reversible in vitro control of protein condensate formation. (a) Confocal microscopy images of heat shock factor 1 (HSF1) and fused in sarcoma (FUS) droplets at 60 min after the addition of crowding agents. For OFF and ON OptoChaperone conditions, samples were irradiated for 5 min with UV (OFF) or blue (ON) light. Samples contain purified protein (HSF1 or FUS with 10% fluorescent-labeled protein) with or without equivalent concentrations of OFF OptoChaperone, ON OptoChaperone, or unmodified chaperone. Droplet formation was triggered using crowding agents: 10% w/v Ficoll (HSF1) and 4% w/v PEG8000 (FUS). Scale bar: 10 μm. (b) Time-course turbidity measurements (600 nm) of HSF1 solutions during three cycles of alternating UV and blue light exposure (5 min each). After 30 min of adding a crowding agent or light irradiation, the next light irradiation was performed for 5 min, and the turbidity was measured again. Data are presented as mean turbidity ± standard deviation (s.d.) from three independent experiments. (c) Normalized turbidity of HSF1, FUS, and TAR DNA-binding protein 43 (TDP-43) solutions at 30 min after initial UV or blue light exposure (first cycle). Values were normalized to turbidity of the group without chaperone (set as 1.0). Bars represent the mean ± s.d. of three independent experiments.

Regulation of protein condensates was also evaluated by the turbidity assays (Figures 3b and S9). In the presence of OptoChaperone after blue light (ON state), the turbidity was maintained at ∼0.2, whereas after UV light irradiation, inducing the OFF state, the turbidity increased to ∼0.5, indicating that the ON state OptoChaperone successfully suppressed HSF1 assembly, whereas the OFF state OptoChaperone allowed assembly. Furthermore, the data showed that the formation and suppression of HSF1 droplets were reversible (Figures 3b and S9). Turbidity decreased after 5 min irradiation with blue light, indicating the suppression of HSF1 droplets. In contrast, turbidity increased after 5 min of UV light irradiation, indicating a reduction in OptoChaperone activity. These reversible effects were observed for up to three cycles, demonstrating the reversible switching of chaperone activity.

Photoswitching of protein condensation was also demonstrated using FUS and TDP-43. Microscopic observations showed that OptoChaperone was also capable of controlling FUS droplets, with significant differences in terms of the size and number of FUS droplets between the ON and OFF states (Figures 3a and S10). The turbidity assay corroborated the microscopic observations, showing significant changes in turbidity after light irradiation (Figures 3c and S11). A turbidity assay was also performed for TDP-43, showing that OptoChaperone is effective in regulating the TDP-43 condensate (Figures 3c and S12).

Collectively, in vitro assays demonstrated that OptoChaperone is effective for HSF1, FUS, and TDP-43 proteins, suppressing droplet formation after blue light, which triggers the ON state of OptoChaperone and allows droplet formation after UV light, which triggers the OFF state of OptoChaperone. Nevertheless, the effects on these proteins could differ owing to variations in the substrate recognition mechanisms of TF. Taking advantage of the broad client recognition properties of TF, OptoChaperone, consisting of TF^PPD‑SBD^, can handle many different disordered proteins.

### OptoChaperone Regulates Protein Condensates in Cells

Subsequently, to evaluate whether OptoChaperone regulates the protein condensate of HSF1 in cells, OptoChaperone was introduced into HeLa cells, and HSF1 condensation after blue/UV light irradiation was observed using confocal microscopy (Figures 4a and S13). After electroporation followed by a 3 h incubation period at 37 °C, the cells were exposed to blue and UV light for 5 min and subsequently treated under heat at 43 °C for 1 h. Endogenous HSF1 was observed using immunostaining. Confocal images of the cells after heat treatment showed that a larger number and size of HSF1 foci were observed for cells containing OFF-state OptoChaperone than for those containing ON-state OptoChaperone (Figures 4b and S14). Evaluation of the foci area corroborated this observation, showing that cells containing OFF-state chaperones induced by UV irradiation possessed a larger area of HSF1 foci than that in those containing ON-state chaperones induced by blue light (Figure 4c). Furthermore, sequential light irradiation experiments demonstrated the dissolution of pre-existing HSF1 condensates by switching the OptoChaperone from OFF to ON state (Figure S15). Note that the UV/blue light irradiations induced a negligible effect on the cells without OptoChaperone (Figure S14).

**Figure 4:**
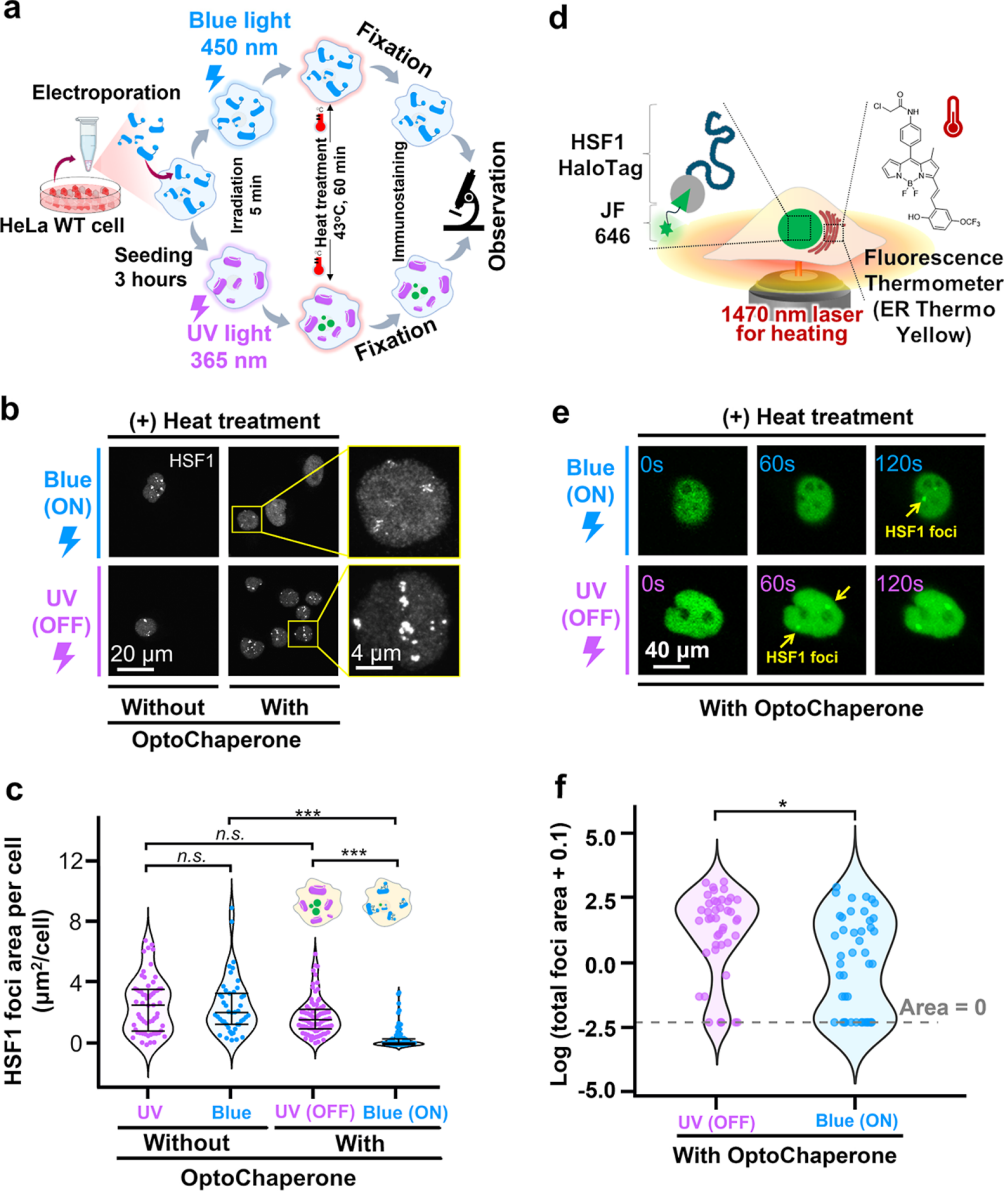
OptoChaperone-mediated control of protein condensates in cells. (a) Schematic of the experimental workflow for observing fixed intracellular foci formation. HeLa cells were electroporated with OptoChaperone, incubated for 3 h at 37 °C, and then exposed to either 5 min of UV or blue light. All groups were subsequently subjected to 60 min heat shock, fixed, immunostained, and observed. See also the Methods Section for electroporation efficiency, and Figure S13 for the characterization of OptoChaperone localization. (b) Representative confocal images of endogenous HSF1 foci formation modulated by OptoChaperone. OptoChaperone-introduced cells were fixed after treatment steps as in (a) and visualized using anti-HSF1 antibody. Scale bar: 20 μm. Enlarged images of the yellow boxed areas are shown. Scale bar: 4 μm. (c) Quantitative analysis of HSF1 foci formation in cells. The violin plot displays the distribution of individual data points (small dots) pooled from three independent experiments, overlaid with the median and 25th–75th percentiles (without: UV *n* = 60; blue *n* = 45; with OptoChaperone: UV/OFF *n* = 82; blue/ON *n* = 62). Statistical significance was determined by the Kruskal–Wallis test followed by Dunn’s multiple comparisons test (n.s., not significant; ***p < 0.001). (d) Schematic illustration of experimental design for the investigation of OptoChaperone in live cells. HeLa cells stably expressing HSF1-HaloTag were stained with JF646 Halo ligand to visualize HSF1, and with an ER-localized fluorescent thermometer to quantify intracellular temperature. A localized heat spot was generated by IR laser heating (1470 nm) of water. (e) Representative time-lapse fluorescence images showing the effect of OptoChaperone activity on HSF1 foci formation (green) in a single living cell during localized heat stress (0–120 s). Blue/ON OptoChaperone (top); UV/OFF OptoChaperone (bottom). An extended time-course with additional time points is provided in Figure S17c. (f) Comparative analysis of heat-induced foci formation using single-cell thermometry in ON versus OFF OptoChaperone (derived from Figure S17a). The violin plot displays distribution of individual data points (small dots) pooled from three independent experiments (OFF *n* = 44; ON *n* = 44). To accommodate cells with no visible foci (area = 0), the total foci area was natural log-transformed after adding a pseudocount of 0.1 [ln­(total Foci area +0.1)]. The dashed line indicates the baseline value corresponding to an area of zero. Statistical significance was determined by the bootstrap two-sample Kolmogorov–Smirnov test (*p < 0.05).

These results are corroborated by the living cell experiments (Figures 4d,e, S16 and 17 and Movie M1). To monitor the real-time dynamics of HSF1 in living HeLa cells, HaloTag-fused HSF1 was expressed and subsequently labeled with Janelia Fluor 646 (JF646) dye. In parallel, intracellular temperature at the single-cell level was analyzed using a fluorescent thermometer (ER thermo yellow) capable of reporting temperature changes as fluorescence intensity (Figure 4d).^51^ Under microscopic observation, a 1470 nm infrared (IR) laser was delivered through the objective lens to generate a micron-sized heat spot within the cell cluture dish by a photothermal effect to activate the motion of water molecules.^52^ Using the fluorescent thermometer, the temperature increase experienced by individual cells was determined at the single-cell level (Figure S16). As a representative example, photothermal heating at 43 °C rapidly induced HSF1 foci formation in the OFF state of the OptoChaperone, whereas this response was clearly delayed and attenuated in the ON state (Figure 4e). Across 40–45 °C, the ON state significantly reduced total foci area compared with OFF, particularly by decreasing large-foci cells at >42 °C (Figures 4f and S17). These observations were highly consistent with in vitro results and highlighted the ability of OptoChaperone to function under cellular conditions.

### Photoswitching of OptoChaperone Determines the Fate of Cells under Stressed Conditions

Building on the regulatory role of OptoChaperone in cells (Figure 4), we evaluated the effect of OptoChaperone activity in cells. Given that the stress response induces the formation of several protein condensates, including nuclear stress bodies containing HSF1 and stress granules containing FUS and TDP43, the photoswitching of OptoChaperone was expected to affect the stress response system. Therefore, we evaluated the cell viability after heat treatment in the absence and presence of ON/OFF-OptoChaperone (Figures 5 and S18). The cell viability of the OFF-OptoChaperone group was comparable to that of the non-OptoChaperone group but significantly higher than that of the ON-OptoChaperone group. The reduced cell viability with ON-OptoChaperone can be attributed to the suppression of the stress-responsive protein condensates as represented by HSF1 condensates (Figure 4), confirming the critical function of these protein condensates for cells to survive under stress conditions.^53^ Notably, cell fate is determined during the heat shock period, followed by a delayed detection of death or senescence-like arrest.^54,55^ This immediate commitment to a specific cellular outcome occurs well within the operational lifetime of the OptoChaperone’s ON and OFF states. These data showed that OptoChaperone not only controls protein condensates but also controls cell fate under stressed conditions, demonstrating its effectiveness in studying the functional importance of protein condensates in the cell.

**Figure 5:**
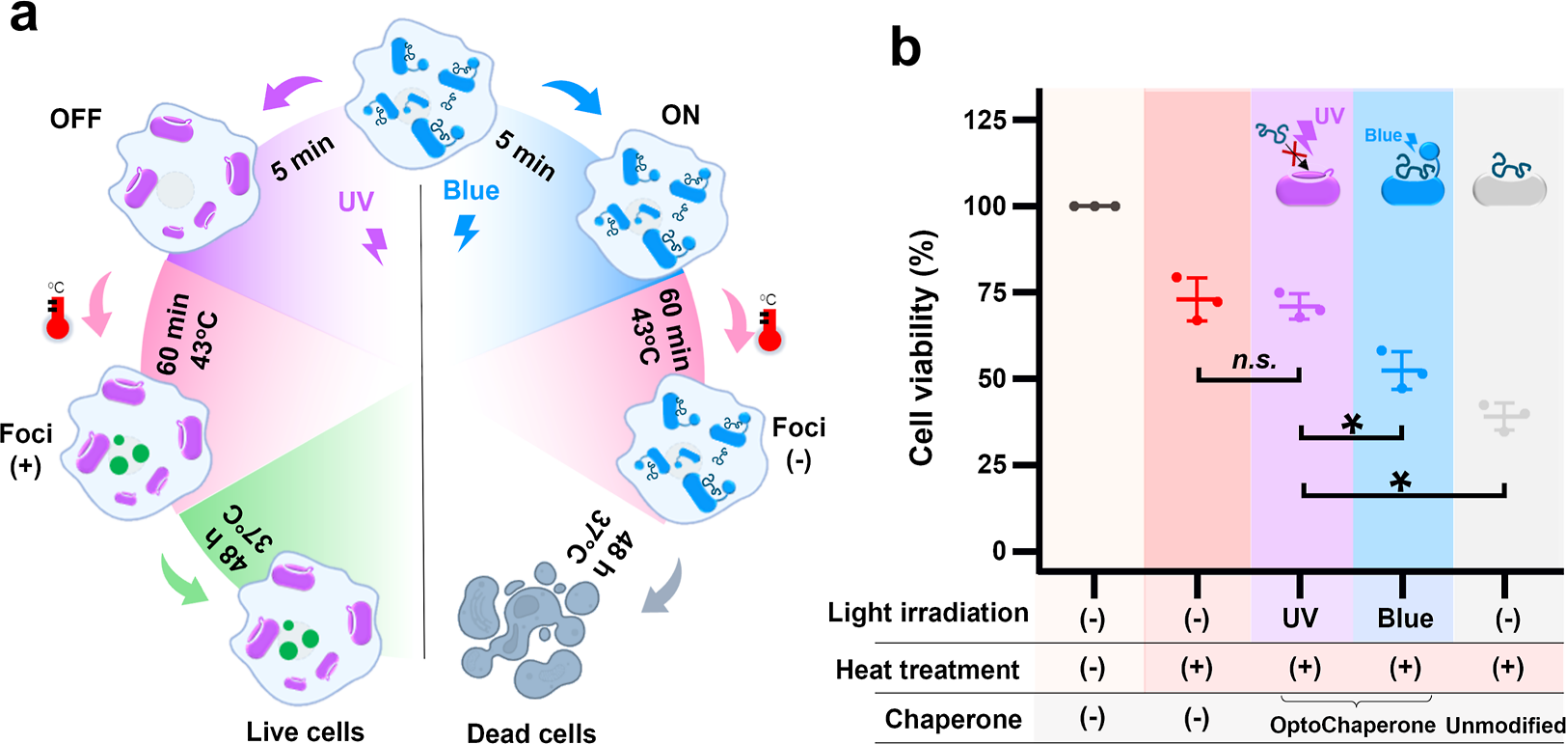
Effect of OptoChaperone on cell viability under heat treatment. (a) Schematic of cell viability assay. HeLa cells electroporated with OptoChaperone were seeded 96-well plates. After adherence, cells were irradiated for 5 min with UV (OFF) or blue (ON) light to switch the state of OptoChaperone immediately before heat treatment. Cells were then subjected to 120 min of heat treatment at 43 °C, followed by a 48 h incubation at 37 °C to assess delayed cytotoxicity. Cell viability was measured using a water-soluble tetrazolium 8 (WST-8) assay after 4 h of incubation with the reagent. (b) Quantitative analysis of cell viability after light irradiation and heat treatment. Viability values were normalized to those of HeLa cells without OptoChaperone and without heat treatment (set as 100%). The plots show each data points, and the mean ± s.d. (*N* = 3). Statistical significance was determined by one-way ANOVA followed by Tukey’s HSD (n.s., not significant; *p < 0.05).

## Discussion

Unlike traditional methods, which rely on altering environmental conditions such as temperature, pH, ionic strength, and molecular crowding to induce protein condensation,^20–22^ OptoChaperone leverages light to exert spatiotemporal control over protein condensates, allowing researchers to induce or reduce protein condensates without disturbing the cellular environment. Rather than modulating the expression levels of target proteins, OptoChaperone enables direct control over their physicochemical state, facilitating the dynamic regulation of assembly and disassembly. This capability will advance our understanding of the functional significance of protein condensation in various biological processes. One of the key strengths of the OptoChaperone is its reversibility. While previous optogenetic tools driving protein assembly often result in irreversible protein condensation,^24–29^ OptoChaperone accelerates the dissociation of the protein condensate, which ensures reversible regulation. Given its unique concept and novel intervention points, OptoChaperone can be combined with other optogenetic tools that induce protein assembly using light. This extends the utility and potential application of optogenetic tools to study protein condensate dynamics and function in cells and in vitro. OptoChaperone offers advantages in controlling protein condensation through light, but the *cis/trans* switching of BSBCA requires near-UV (UVA, 365 nm) light, which may raise concerns about potential cellular damage.^56,57^ Notably, UVA is distinct from shorter-wavelength UVB/UVC that is more strongly DNA-damaging. Consistent with this, we observed minimal effects of near-UV and blue light on cell viability under our experimental conditions (Figure S18a). Nevertheless, this issue will be adequately addressed in future studies by using azobenzene derivatives responding to visible or IR light^58^ that are less harmful to cells. To further facilitate in vivo applications, the biohybrid system could be delivered using sophisticated nanocarrier systems.^57,59,60^

Here, we developed a nonselective OptoChaperone using a promiscuous chaperone TF, which offers a versatile, broad-spectrum platform to regulate multiple distinct proteins without the need to redesign the core module for each specific target. Under heat stress conditions, the cellular phenotypes observed with OptoChaperone are predominantly driven by the disruption of HSF1 condensate. HSF1 is the most abundant and functionally dominant factor in the heat stress response, and its failure leads to cell death.^45^ Thus, although OptoChaperone may interact with other condensates, the biological outcome under this specific stress is context-dependent and dominated by HSF1. For applications requiring greater target selectivity, light irradiation to specific subcellular regions could achieve spatial selectivity. Additionally, the same strategy can be applied to other chaperones, including those that recognize specific targets, by fusing a lid protein labeled with BSBCA because many chaperones share the ability to recognize unfolded hydrophobic regions of the proteins. This lid-based strategy opens up new applications for this tool. The application of OptoChaperone is not limited to protein condensate studies but is also beneficial in the functional analysis of a chaperone in the cell. Given the increasing interest in chaperones related to diseases involving protein aggregation, OptoChaperone will be an important tool in broad research fields, including neurodegenerative diseases. OptoChaperone provides a platform for developing therapeutic strategies targeting pathological protein condensates, paving the way for new research directions and clinical applications.

## Methods

### Plasmids Construction, Protein Expression, and Purification

Recombinant plasmids were constructed at Tokushima University and Hokkaido University. GB1 (T25C/K28A/V29A/Q32A/Y33A/D36C) was expressed in the pET21d­(+) vector. Mutations were introduced into GB1, and a stop codon was inserted behind the His-tag to ensure expression of only the mutated GB1 and His-tag. GB1-His_6_-TEV-GB1-(GS)_5_-TF^PPD‑SBD^: GB1-(GS)_5_-TF^PPD‑SBD^ was cloned into the pET21d­(+) vector and fused with a GB1-His_6_ tag at the N-terminal TEV protease cleavage site. GB1-His_6_-HSF1: a codon-optimized recombinant plasmid encoding HSF1 was synthesized by Kawagoe et al.^44^ HSF1 constructs were cloned into the pET21b vector (Cat No. 69741-3CN, Novagen) and fused to a GB1-His_6_ tag at the HRV3C N-terminal PreScission protease cleavage site. MBP-TEV-FUS: FUS proteins were expressed in the pMAL-TEV vector using the MBP fusion constructs. MBP-TEV-TDP-43: TDP-43 proteins were expressed using MBP fusion constructs in the pMAL-TEV vector.

All the recombinant proteins were expressed individually in BL21­(DE3) cells. All the expression constructs were introduced into BL21­(DE3) cells. With respect to the samples, cells were grown in Luria–Bertani medium at 37 °C in the presence of ampicillin (50 μg mL^–1^). Subsequently, protein expression was induced by adding 0.5 mM isopropyl-β-D-1-thiogalactopyranoside (IPTG) at OD_600_ ∼0.6, followed by 12–16 h of incubation at 18 °C. The cells were harvested at OD_600_ ∼3.0, resuspended in lysis buffer containing 50 mM Tris–HCl (pH 8.0) and 500 mM NaCl, and stored at −80 °C. GB1-His_6_ and GB1-His_6_-TEV-GB1-(GS)_5_-TF^PPD‑SBD^ were introduced into BL21­(DE3) cells for recombinant protein expression. The cells were disrupted using a sonicator and centrifuged at 18,000 rpm for 30 min. The supernatant containing the target protein was purified using a Ni-NTA Sepharose column (Cat No. 30230; QIAGEN, Germany). After washing (using 50 mM Tris pH 8.0, 500 mM NaCl, 50 mM Tris pH 8.0, 150 mM NaCl, and 15 mM imidazole), the proteins were eluted with an elution buffer (50 mM Tris pH 8.0, 150 mM NaCl, and 400 mM imidazole). TEV protease was used to remove the purification tag (GB1-His-TEV), followed by overnight dialysis (50 mM Tris HCl, 150 mM NaCl, 4 mM 2 ME, pH 8.0) at 4 °C. The GB1-(GS)_5_-TF^PPD‑SBD^ protein was further purified using a Ni-NTA resin (Cat No. 30230; QIAGEN, Germany). Size-exclusion chromatography was performed using a HiLoad 26/600 Superdex 200 pg column (Cat No. 10250637, Cytiva, Sweden; Buffer 50 mM Tris–HCl, 100 mM NaCl, pH 8.5), and the purified protein was concentrated. The final concentration (1 mM) was used for BSBCA labeling, followed by anion-exchange purification (Resource Q 6 mL column, Cat No. 10308898; Cytiva, Sweden) with 0–50% linear gradient elution (30 column volumes) in buffer A (50 mM Tris–HCl, pH 8.5) and buffer B (50 mM Tris–HCl, 1 M NaCl, pH 8.5). The purified protein was collected and stored at −80 °C. The expression and purification of HSF1,^44^ FUS,^33^ and TDP-43 proteins^42^ were performed according to a previous study.

For mammalian cell expression, the human HSF1 cDNA was amplified and fused with a HaloTag in the PB533-Halo vector.^61^

### Generation of HeLa-HSF1-Halo Cells

For piggyBac transposon system-mediated stable introduction of the HSF1-Halo into HeLa cells, we followed the methods described previously.^62^ Briefly, 4 μg PB533-HSF1-Halo and 1 μg pcDNA3-mPB were mixed with 15 μL FuGENE 4K (Cat No. E5911, Promega, Madison, WI, USA) added on to a 100 mm dish following the manufacturer’s instructions. Transfected cells were selected by incubation in medium containing 400 μg/mL G-418 disulfate aqueous solution (Cat No. 09380-44, Nacalai Tesque, Kyoto, Japan). Four days after positive selection, HeLa-HSF1-Halo cells were trypsinized and incubated with 100 nM JF646 HaloTag Ligand (Promega) for 45 min, then HSF1-Halo-positive population was collected using the SH800S cell sorter (Sony Biotechnology).

### Protein Isotope Labeling for NMR Studies

^15^N-isotopically labeled GB1 for NMR studies was prepared by growing the cells in a minimal (M9) medium containing ^15^NH_4_Cl (1 g liter^–1^). Protein expression was induced by adding 0.5 mM IPTG at OD_600_ ∼0.6, followed by 12–16 h of incubation at 18 °C. The cells were harvested at OD_600_ ∼2.0, resuspended in lysis buffer containing 50 mM Tris–HCl (pH 8.0) and 500 mM NaCl, and stored at −80 °C. For the isotope labeling of TF^PPD‑SBD^ and GB1-(GS)_5_-TF^PPD‑SBD^, the M9 medium containing ^15^NH_4_Cl and [^2^H,^12^C]-glucose in 99.9% D_2_O was used. For the production of U-[^2^H], Ile-δ1-[^13^CH_3_], Val, Leu-[^13^CH_3_,^12^CD_3_], Met-[^13^CH_3_], and Ala-[^13^CH_3_] TF^PPD‑SBD^ and GB1-(GS)_5_-TF^PPD‑SBD^, alpha-ketobutyric acid [methyl-^13^CH_3_] (50 mg liter^–1^), alpha-ketoisovaleric acid [monothyl-^13^CH_3_,^12^CD_3_] (85 mg liter^–1^), [^13^CH_3_]-Met (50 mg liter^–1^), and [D_2_, ^13^CH_3_]-Ala (50 mg liter^–1^) were added to the culture 30 min before adding IPTG. Protein expression was induced by adding 0.5 mM IPTG at OD_600_ ∼0.6, followed by 12–16 h of incubation at 18 °C. The cells were harvested at OD_600_ ∼2.0, resuspended in lysis buffer containing 50 mM Tris–HCl (pH 8.0) and 500 mM NaCl, and stored at −80 °C. The samples were purified, and BSBCA labeling was performed using the same procedure as that used for culturing in a Luria–Bertani medium.

### Introduction of BSBCA into GB1 or GB1-(GS)_5_-TF^PPD‑SBD^

In a nitrogen atmosphere, a glovebox was used to introduce BSBCA into a GB1 or GB1-(GS)_5_-TF^PPD‑SBD^ solution (1 mM). TCEP (2 mM) was then added, and the mixture was stirred at room temperature for 30 min. BSBCA (5 mM) was then added and stirred at 25 °C for 6 h. The reaction solution was recovered, stored at 4 °C, and separated using an anion exchange column with a linear gradient elution (Resource Q 6 mL, Cat No. 10308898, Cytiva, Sweden). The BSBCA-GB1 or BSBCA-GB1-(GS)_5_-TF^PPD‑SBD^ (OptoChaperone) fractions were concentrated and frozen in liquid nitrogen. According to a previous study,^37^ the *cis* and *trans* populations were determined from absorbance values. The *trans* concentration was calculated using eq 1, assuming a negligible *cis* absorbance at 365 nm. The *cis* concentration was obtained using eq 2, while the BSBCA in *trans*- and *cis*- populations were determined using eqs 3 and 4, respectively.

1 $$C_{trans}=\frac{A_{365}}{\varepsilon_{trans,365}}$$

2 $$C_{cis}=\frac{A_{450}-\left(C_{trans}\times \varepsilon_{trans,450}\right)}{\varepsilon_{cis,450}}$$

3 $$trans-population\left(\%\right)=\frac{C_{trans}}{C_{trans}+C_{cis}}\times 100\%$$

4 $$cis-population\left(\%\right)=\frac{C_{cis}}{C_{trans}+C_{cis}}\times 100\%$$

where *C*_*trans*_: concentration of *trans*-population; *C*_*cis*_: concentration of *cis*-population, *A*_365_: absorbance of azGB1 at 365 nm; *A*_450_: absorbance of azGB1 at 450 nm; ε_*trans*,365_: extinction coefficient of *trans* at 365 nm (=24,000 M^–1^ cm^–1^); ε_*trans*,450_: extinction coefficient of *trans*-population at 450 nm (=2100 M^–1^ cm^–1^); ε_*cis*,450_: extinction coefficient of *cis*-population at 450 nm (=2900 M^–1^ cm^–1^).

### Matrix-Assisted Laser Desorption/Ionization-Time-of-Flight Mass Spectrometry

The samples were mixed with a saturated solution of sinapinic acid in 50% acetonitrile and 0.1% trifluoroacetic acid at a 1:1 ratio (v/v). A 1.0 μL aliquot of the mixture was spotted onto a MALDI target plate and air-dried at room temperature. Mass spectrometry analysis was performed using an Autoflex Speed (Bruker) with a Smartbeam II. Ionization was performed in positive linear mode with an acceleration voltage. Mass spectra were acquired over an *m*/*z* range of 5–20 kDa using Bruker Daltonics flex analysis. Mass spectra were analyzed using Bruker Daltonics Flex Analysis to identify peaks corresponding to biomolecules of interest. The signal intensities and peak distributions were compared between the experimental groups to assess potential biomarker differences (Figure S19).

### NMR Experiments

NMR samples were prepared in 20 mM KPi (pH 7.0), 100 mM KCl, 4 mM βME, 0.5 mM EDTA, 0.05% NaN_3_, and 7% D_2_O. Protein concentrations were 0.02 mM azGB1, 0.3 mM TF^PPD‑SBD^, and 0.5 mM azGB1-TF^PPD‑SBD^. NMR experiments were performed using a Bruker Avance Neo 800 MHz NMR spectrometer with cryogenic probes and a Bruker Avance III 500 MHz NMR spectrometer. The experiments were performed at 25 °C for azGB1 and at 22 °C for TF^PPD‑SBD^ and azGB1-TF^PPD‑SBD^. Following initial NMR acquisition, UV/blue light irradiation was applied for 5 min. Spectra were processed using the NMRPipe program,^63^ and data analysis was performed using Olivia (https://github.com/yokochi47/Olivia). The chemical shift perturbations of the amide moiety were normalized according to the equation below (eq 5)­

5 Δδ=(Δδ(H1))2+(Δδ(N15)/5)2

The signal assignment information for TF^PPD‑SBD^ was obtained using BMRB entries 19,836 and 19,837.^34^

### Microscopic Observation

For HSF1 droplet formation, a solution containing 18 μM HSF1, 2 μM GFP-HSF1, 20 μM OptoChaperone, and 10 mM DTT was prepared by mixing all components with HSF1 purification buffer (25 mM HEPES/KOH, 150 mM KCl, pH 7.2) to a final volume of 12.5 μL. The solution was incubated at room temperature for 10 min before adding Ficoll400 (10% w/v, final volume 25 μL) to induce droplet formation. For MBP-FUS droplets, a solution of 4.5 μM FUS, 0.5 μM CF488A-FUS, 10 μM OptoChaperone, and 10 mM dithiothreitol (DTT) was prepared using FUS purification buffer (20 mM HEPES, 1 M NaCl, 10% glycerol, 2 mM DTT, pH 7.4) to a final volume of 40 μL, followed by incubation for 10 min. PEG8000 (4% w/v, final volume 50 μL) was added to trigger droplet formation. Microscopic observations were conducted immediately using a laser scanning microscope equipped with a 40× objective lens. Light irradiation (5 min) for OptoChaperone activation was performed under the same conditions as the absorption spectrum measurements using a light guide positioned between an aluminum foil-shaded observation dish and the microscope. The radius and area occupancy ratio of the droplets in the image were measured using the ImageJ software, where the area-occupancy ratio is the proportion of the area occupied by each droplet within the field of the microscope.

### Turbidity Assay

Turbidity assays were performed using a V-730 spectrophotometer (V-730 Bio Spectrophotometer, JASCO, Japan) at 25 °C with an initial blank measurement in the corresponding purification buffer after 5 min of light irradiation. HSF1 (20 μM), FUS (5 μM), and TDP-43 (10 μM) solutions were prepared in their respective purification buffers with OptoChaperone (equal molar ratio) and DTT (10 mM) and incubated at room temperature for 30 min. Droplet formation was induced by adding Ficoll (10% w/v) to HSF1 or PEG8000 (4% w/v for FUS and 8% w/v for TDP-43), followed by pipet mixing. The solutions (400 μL) were transferred to a quartz microcell, and turbidity (600 nm) was recorded every 30 s for 30 min. After the measurement, the samples were irradiated with light, shielded with aluminum foil, and remeasured under identical conditions.

### Fluorescence Anisotropy Assay

The binding affinity between ON or OFF OptoChaperone and HSF1 was characterized using a fluorescence anisotropy assay. HSF1 was labeled with CF568 maleimide (Cat No. 92024, Biotium, Fremont, CA, USA). Immediately before the following incubation, OptoChaperone was activated by exposure to blue light for 5 min (ON) or inactivated by exposure to UV light for 5 min (OFF). A constant concentration of CF568-labeled HSF1 (1 μM HSF1-CF568) were incubated with increasing concentrations of ON or OFF OptoChaperone in a buffer containing 25 mM HEPES/KOH, 150 mM KCl, pH 7.2 for 5 min at 25 °C. Fluorescence Anisotropy measurements were performed using a spectrofluorometer (FP-8350, Jasco, Japan) and manual polarizers (FDP-243, Jasco, Japan). The excitation and emission wavelengths were set at λ_ex_ = 562 nm and λ_em_ = 583 nm, respectively.

The fluorescence anisotropy (*r*) at each titration point was calculated from the vertical (*I*_VV_) and horizontal (*I*_VH_) emission intensities recorded with vertically polarized excitation light, according to the following equation

6 $$r=\frac{I_{VV}-G\times I_{VH}}{I_{VV}+2G\times I_{VH}}$$

where *G* is the grating correction factor (*G* = *I*_HV_/*I*_HH_) determined from the vertical (*I*_HV_) and horizontal (*I*_HH_) emission intensities recorded using horizontally polarized excitation light.

The change in anisotropy, Δ*r*, was calculated as Δ*r* = *r*_obs_ – *r*_free_, where *r*_obs_ is the anisotropy (*r*) observed at each OptoChaperone concentration and *r*_free_ is the anisotropy of 1 μM HSF1-CF568 in the absence of OptoChaperone. To ensure data accuracy, the total fluorescence intensity was monitored to ensure the absence of significant protein-induced quenching. To ensure data accuracy, all measurements were corrected for background fluorescence using a buffer-only control.

### Cell Culture for Immunofluorescent Staining

HeLa cells were obtained from RIKEN BRC cell bank (RCB007). The HeLa cells were cultured and maintained at 37 °C and 5% CO_2_ in Dulbecco’s modified eagle’s medium (DMEM) (Cat No. 08458-45; Nacalai Tesque) supplemented with 10% fetal bovine serum (FBS) (Cat No. S-FBS-NL-015; Serana, Brandenburg, Germany). Once the cells proliferated to cover the dish, they were passaged every 2–3 days. The medium was removed, and the cells were washed twice with phosphate-buffered saline (PBS; 10 mM Na_2_HPO_4_, 1.76 mM KH_2_PO_4_, 137 mM NaCl, 2.7 mM KCl). Trypsin-EDTA solution (Cat No. 32777-44; Nacalai Tesque) diluted 0.025% (w/v) in PBS was added to detach the cells, followed by incubation for 5 min. Trypsin was neutralized with 8 mL of DMEM, and the suspension was centrifuged (100 rpm for 3 min). The supernatant was removed, and the pellets were resuspended in 2 mL of DMEM. A 15 μL sample was used for counting in a hemocytometer, and cell density was calculated before reseeding in fresh DMEM. The cell suspension was adjusted so that the cell density per dish was 6 × 10^5^, and the cells were seeded.

### Fluorescence Labeling for OptoChaperone

To track the localization of OptoChaperone within cells, it was fluorescently labeled with ATTO 633 NHS-Esters (Cat No. AD633-35; ATTO-TEC GmbH, Germany) using “ATTO 633 Protein Labeling Kit”.^64^

### Cellular Introduction of OptoChaperones Using Electroporation

HeLa cells were harvested, and an aliquot containing 1.0 × 10^6^ cells were transferred to a 1.5 mL tube, followed by centrifugation at 1000*g* for 3 min. The supernatant was removed. The cell pellet was then washed twice with 1 mL of electroporation buffer and mixed with 100 μL of 500 μM OptoChaperone solution (50% fluorescence labeling). The suspension was transferred to an electroporation cuvette, warmed to 37 °C, and subjected to electroporation using a NEPA21 superelectroporator (Nepa Gene, Chiba, Japan). The poring pulse was set at 100 V for 15 ms, and the transfer pulse was set at 20 V for 50 ms with alternating polarity. Finally, 200 μL of prewarmed DMEM was added for recovery. The electroporation efficiency was determined to be 98.2% (calculated as 336 ATTO-633 positive cells out of 342 DAPI-stained nuclei), confirming that OptoChaperone was successfully introduced into most cells for subsequent analyses.

### Light Irradiation

For UV exposure, a Xenon light source (model: MAX-303, Asahi Spectra, Japan) was configured to emit monochromatic near-UV light isolated using a 365 nm bandpass filter at its maximum power output for 5 min. Subsequently, the light source was transitioned to emit blue light, filtered using a 450 nm bandpass filter, again at maximum power output, for an additional 5 min exposure period. The distance between the MAX-303 light source and sample was kept constant throughout both irradiation steps to ensure a consistent energy fluence. The illumination distance to the cells was 200 mm, resulting in a near-UV irradiance of 1.65–1.88 mW/cm^2^ at 365 nm. With a 5 min exposure, the received dose ranged from 0.495 to 0.564 J/cm^2^, which is well below the 1.6 J/cm^2^ threshold for photobiological damage in HeLa cells.^65^

### Heat Treatment

For immunostaining experiments, cells with introduced OptoChaperones were subjected to heat stress to induce droplet formation on endogenous HSF1. First, the cells were seeded onto poly l-lysine-coated coverslips by floating them in a coating solution, incubated for 15–30 min, and washed with PBS. A cell suspension with OptoChaperones was then seeded onto the coated coverslips in a six-well plate with DMEM, achieving a density of 2.5 × 10^5^ cells per well, and incubated at 37 °C for 4 h. For protein condensate formation on HSF1, the medium was replaced with a preheated 43 °C medium (with or without 0.1% w/v 1,6-hexanediol), and the plate was incubated at 43 °C. After 60 min, cells were washed with PBS, fixed with 4% paraformaldehyde for 15 min, washed again, and stored in PBS at 4 °C in the dark.

### Immunofluorescent Staining and Observation

The treated cells were washed twice with PBS at room temperature (RT) and fixed with a 4% paraformaldehyde phosphate buffer solution (Cat No. 09154-58; Nacalai Tesque) for 15 min. The fixed cells were washed four times with PBS at RT and permeabilized with PBS containing 0.1% TritonX-100 (Cat No. 12967-32; Nacalai Tesque) for 15 min and then blocked with PBS containing 2% FBS for 1 h at RT. Next, the cells were incubated at 4 °C overnight with antibodies against HSF1 (Cat No. 4356; Cell Signaling Technology, Danvers, MA, USA; 1:500) and HSP70 (Cat No. sc-24; Santa Cruz Biotechnology, Santa Cruz, CA, USA; 1:200) diluted in PBS containing 2% FBS. Following incubation, the cells were washed thrice with PBS at RT and then incubated at 4 °C for 1 h with CF488A-conjugated donkey antirabbit IgG (Cat No. 20015-1; biotium; 1:2000) and CF568-conjugated goat antimouse IgG (Cat No. 20101-1; biotium; 1:2000) as secondary antibodies, diluted in PBS containing 2% FBS. Finally, the cell nuclei were stained with 4′,6-diamidino-2-phenylindole (DAPI; Cat No. 19178-91; Nacalai Tesque; 1:10,000). Then, immunostained cells were encapsulated using Fluoro-KEEPER Antifade Reagent, sealed with nail polish, and stored at 4 °C to preserve fluorescence. Fluorescent images were obtained using a confocal microscope (FV1200, Olympus) equipped with a 60× silicon oil immersion objective lens (UPLSAPO60XS2, Olympus; NA 1.30). The total foci area in each cell in the image was measured using software ImageJ/Fiji^66^ (also see below “Total Foci Quantification and Analysis” Section). To ensure the analysis of a representative cell population and minimize variability due to HeLa cell heterogeneity (e.g., ploidy differences), cells were selected based on a nuclear area of 75–150 μm^2^ and a detectable OptoChaperone signal. Cells with abnormal morphology were excluded. All quantitative data were obtained from three independent experiments.

### Total Foci Quantification and Analysis

To quantify the total foci area in each cell, image stacks were processed and analyzed using the open-source software ImageJ/Fiji.^66^ Initially, all images were calibrated to a physical scale using the metadata scaling factors (1 pixel = 0.088 μm). 12 bit grayscale images were processed to isolate the channel containing the specific fluorescent foci. To minimize background noise and uneven illumination, a “rolling ball” background subtraction was applied with a radius of 60 pixels. Subsequently, a global thresholding method was employed to convert the grayscale image into a binary mask. A fixed threshold value of 65 (determined based on preliminary analysis of signal-to-noise ratios) was consistently applied to all images to ensure comparability. The Watershed algorithm was then used to separate touching foci. Finally, the “analyze particles” function was used to measure the area of detected foci. To exclude nonspecific noise and artifacts, particles smaller than 0.01 μm^2^ were excluded from the analysis. Measurements were restricted to the nuclear region of interest (ROI) defined by DAPI staining for each selected cell.

For the analysis of HSF1 foci formation in live-cell imaging, the total foci area was natural log-transformed with a pseudocount of 0.1, calculated as ln­(total foci area +0.1), to properly include cells where no foci were formed (area = 0), to account for the variable and dynamic nature predicted for real-time observations involving short thermal stress.^67^ Because the transformed data exhibited a bimodal distribution and contained a high frequency of identical baseline values (ties), conventional parametric tests and standard rank-sum tests were deemed unsuitable. Instead, we employed a bootstrap two-sample Kolmogorov–Smirnov (KS) test^68^ to compare the overall probability distribution shapes between the ON and OFF conditions. The bootstrap KS test, using 1000 resamples, was performed using the “Matching” package in R.^69^ A *p*-value of <0.05 was considered statistically significant. For comparisons within specific temperature bins, the bootstrap KS test was applied to each bin separately. To account for multiple comparisons, the resulting *p*-values were adjusted using the Holm–Bonferroni method.

### Cell Viability Assay

Wild-type HeLa cells (with OptoChaperone via electroporation) were seeded at a density of 16,000 cells/well in a 96-well collagen type I (Cat No. 2962401; AGC Techno (Iwaki) Glass Co., Shizuoka, Japan) coated microplate. Each well contained 100 μL of DMEM. Cells were allowed to adhere for 2 h at 37 °C and 5% CO_2_. Following adherence, cells underwent either UV or blue light irradiation for 5 min to switch the state of OptoChaperone. Immediately after irradiation, cells were subjected to heat treatment at 43 °C for 120 min. Subsequently, cells were incubated at 37 °C and 5% CO_2_ for 48 h to allow cell viability phenotypes to manifest, with fresh DMEM introduced after 24 h. After the 48 h incubation, 100 μL of DMEM (prewarmed to 37 °C) containing 10% v/v WST-8 reagent (Cat No. 260-96162; Kishida Chemical Co., Osaka, Japan) was added to each well. The plate was then incubated for an additional 4 h. The absorbance was measured at 450 nm using a microplate reader to assess cell viability.

For 1,6-hexanediol treatment control, cells were incubated in the presence of 0.1% w/v 1,6-hexanediol at 43 °C for 120 min (heat treatment). After exposure to heat stress treatment, the cells were maintained at 37 °C and 5% CO_2_ for 48 h, with fresh DMEM introduced after 24 h. After the 48 h incubation, 100 μL of DMEM (prewarmed to 37 °C) containing 10% v/v WST-8 reagent was added to each well. The plate was then incubated for an additional 4 h. The absorbance was measured at 450 nm using a microplate reader to assess cell viability.

### Cell Culture and Electroporation for Live Cell Imaging

HeLa cells stably expressing HaloTag fused heat shock factor 1 (HSF1-Halo) were maintained at 37 °C under 5% CO_2_ in DMEM supplemented with 10% FBS. Cells were passaged routinely before experiments. For intracellular delivery of OptoChaperone, cells were electroporated. Briefly, cells were detached, resuspended in 10 mL of DMEM, and counted using a Fuchs-Rosenthal type hemocytometer. Cell density was adjusted based on manual counting (approximately 60 cells per square), and the suspension was centrifuged to collect the cell pellet. The pellet was resuspended in 300 μL of Opti-MEM (Thermo Fisher Scientific). A 50 μL aliquot of the resulting cell suspension was mixed with 50 μL of OptoChaperone solution. The 100 μL cell OptoChaperone mixture was transferred to a 2 mm gap electroporation cuvette (BEX Co., Ltd.; working volume 40–400 μL) and electroporated using a CUY21EDIT II pulse generator (BEX Co., Ltd.) with the following parameters: poring pulse voltage, 175 V; poring pulse duration, 5.0 ms; poring pulse interval, 50.0 ms; transfer pulse voltage, 50 V; transfer pulse duration, 50.0 ms; number of transfer pulses, 5; capacitance, 940 μF. Immediately after electroporation, cells were transferred onto glass-bottom dishes (Iwaki; No. 1 cover glass, φ 12 mm) precoated with 5 μL of Cellmatrix Type I-C (3 mg mL^–1^). Cells were allowed to attach for 5 h at 37 °C in a 5% CO_2_ incubator, then fluorescently labeled.

### Live-Cell Microscopic Imaging with Photothermal Heating

After the delivery of OptoChaperone, HSF1-Halo-expressing cells were labeled with 50 nM JF646 HaloTag ligand for 30 min, followed by staining with 500 nM fluorescent thermometer for an additional 30 min at 37 °C under 5% CO_2_. Cells were subsequently washed with 1 mL of PBS and transferred to 2 mL of phenol red-free DMEM supplemented with HEPES for live-cell imaging. Live cell fluorescence imaging was performed using an Olympus IX83 inverted confocal microscope equipped with an FV12-FD detector and an oil immersion objective lens (PLAPON 60×, NA = 1.42). Image acquisition and microscope control were carried out using FV10-ASW 4.2 software (Olympus). Fluorescent thermometer and JF646 were excited using 559 and 635 nm lasers, respectively. Emission signals were separated using an SDM640 dichroic mirror and collected through BA575-620 and BA655-755 band-pass emission filters for ER Thermo yellow and JF646, respectively. Z-stack images were acquired by sequential optical sectioning along the *z*-axis across the nucleus, collecting three optical slices with a *z*-step interval of 0.2–0.3 μm between consecutive focal planes. The three slices were processed as a stack in ImageJ/Fiji and combined by average intensity projection.

A IR laser (1470 nm; MDL-MD-1470-2W, Changchun New Industries Optoelectronics Tech, China) was incorporated into the FV1200 confocal microscope for localized cellular heating. The optical path included a 690/808/855/1030 nm dichroic mirror. Laser power delivered to the sample through a 60× objective lens was measured using a Vega power meter fitted with a 10A-V1.1-SH sensor (Ophir Photonics). Continuous IR irradiation was applied for 2 min to induce localized heating. For experiments evaluating temperature dynamics following HSF1 foci formation, Fluorescence intensity of the fluorescent thermometer was recorded during 20 s of continuous IR laser irradiation. Fluorescence intensity changes were converted to temperature increment values using a previously established calibration (temperature sensitivity: 3.9% °C^–1^).^51^

## Supplementary Material





## Data Availability

The data underlying this study are available in the published article and its Supporting Information.

## Notes

Movie M1 (MP4)
Supporting Information: Absorption spectrum, copies of ^1^H–^13^C and ^1^H–^15^N spectra, microscopic observation, turbidity assay, cell viability assay, fixed and living cell experiments (PDF)
