# Supplementary material for: OptoChaperone—A Biohybrid Tool for Regulating Protein Condensates in Cells and In Vitro
Source: J Am Chem Soc. 2026 Apr 20;148(16):17429–42. doi: 10.1021/jacs.6c04074 (PMC13133789; doi:10.1021/jacs.6c04074)
Supplement: Supplementary file 2 [file ja6c04074_si_002.pdf]

Supporting information for

OptoChaperone – A biohybrid tool for regulating protein condensates in cells and in vitro

Do Thanh Tuan[1,2,#], Motonori Matsusaki[3,4,#], Honoka Ota[5], Soichiro Kawagoe[3,4], Hettimudalige Dilini Nisansala[6], Munehiro Kumashiro[3], Noriyoshi Isozumi[7], Hiroyuki Kumeta[8], Yohei Kono[9], Takeshi Shimi[9], Koichiro Ishimori[5,10], Eiichiro Mori[7], Satoshi Arai[9], Tomohide Saio[2,3,4]*

[1] Department of Physiology, Hanoi Medical University, Hanoi 100000, Vietnam

[2] Graduate School of Medicine, Tokushima University, Tokushima, Tokushima 770-8503, Japan

[3] Institute of Advanced Medical Sciences, Tokushima University, Tokushima, Tokushima 770-8503, Japan

[4] Institute of Photonics and Human Health Frontier, Tokushima University, Tokushima, Tokushima 770-8501, Japan

[5] Graduate School of Chemical Sciences and Engineering, Hokkaido University, Sapporo, Hokkaido 060-8628, Japan

[6] Graduate School of Frontier Science Initiative, Division of Nano Life Science, Kanazawa University, Kakuma-machi, Kanazawa 920-1192, Japan.

[7] Department of Future Basic Medicine, Nara Medical University, Kashihara, Nara 634-8521, Japan

[8] Faculty of Advanced Life Science, Hokkaido University, Sapporo, Hokkaido 001-0021, Japan

[9] Nano Life Science Institute (WPI-NanoLSI), Kanazawa University, Kakuma-machi, Kanazawa 920-1192, Japan.

[10] Department of Chemistry, Faculty of Science, Hokkaido University, Sapporo, Hokkaido 060-0810, Japan

[#] These authors contributed equally.

***Corresponding author**

Tomohide Saio, Graduate School of Medicine, Tokushima University, Tokushima, Tokushima 770-8503, Japan; Division of Molecular Life Science, Institute of Advanced Medical Sciences, Tokushima University, Tokushima, Tokushima 770-8503, Japan; Institute of Photonics and Human Health Frontier, Tokushima University, Tokushima, Tokushima 770-8501, Japan; Email: saio@tokushima-u.ac.jp; Tel. +81-88-633-9149; Fax. +81-88-633-9145.

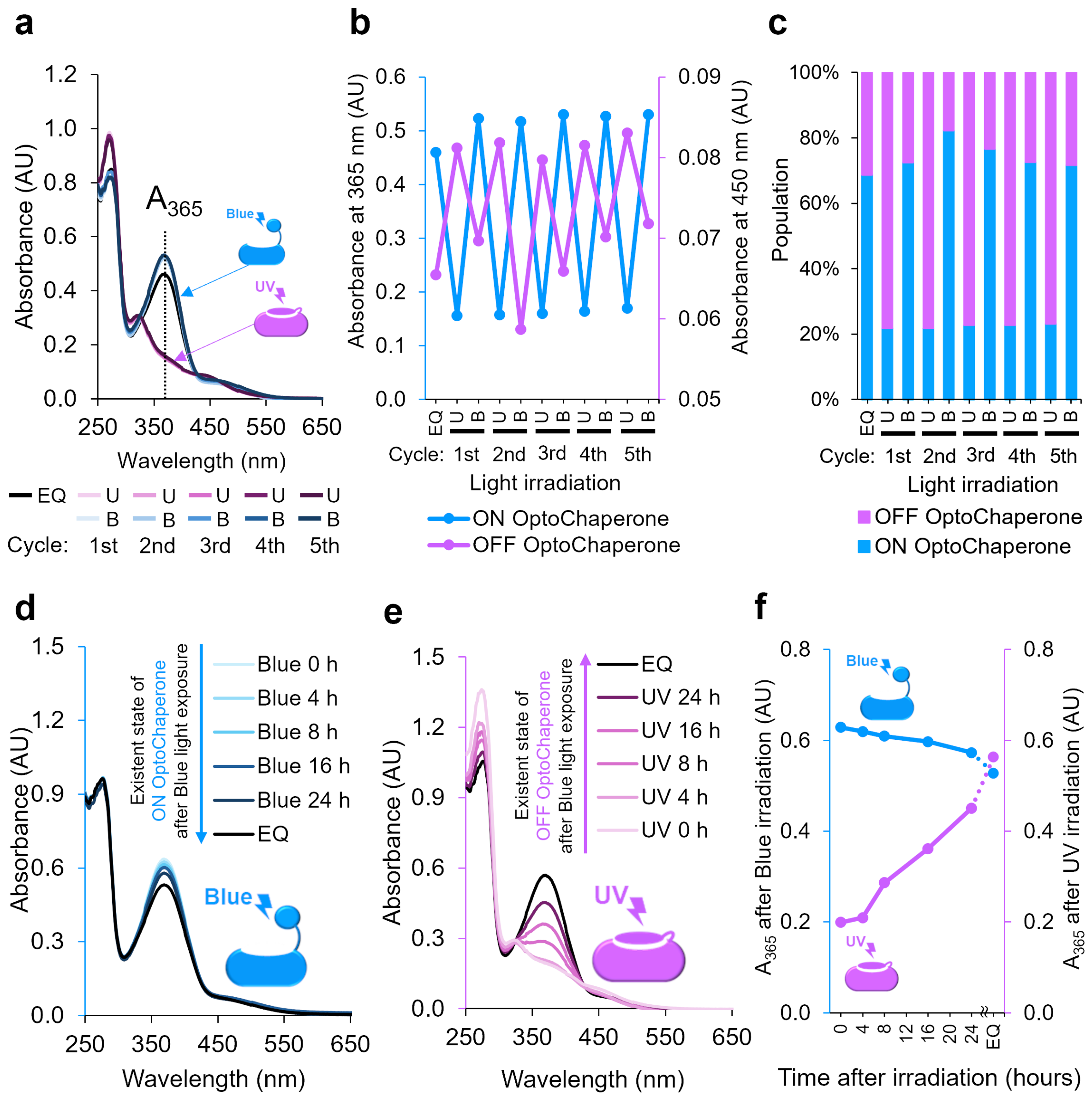

Figure S1. Conformational change and lifespan of OptoChaperone after light irradiation. **(a)-(c)** Conformational change of OptoChaperone after light irradiation. **(a)** Absorption spectrum of OptoChaperone measured after alternately irradiating blue light and UV light for 5 min. **(b)** Change in absorbance at 365 nm and 450 nm when UV and blue light are irradiated alternately. **(c)** The population of the ON and OFF OptoChaperone after 5 min of light irradiation. **(d)-(f)** Lifespan of OptoChaperone after light irradiation**. (d)** Absorption spectrum measured after 5 min of blue light irradiation in dark. **(e)** Absorption spectra measured after 5 min of UV light irradiation in dark. **(f)** Lifespan of ON and OFF OptoChaperone after light

irradiation in a dark environment. (EQ: Thermal Equilibrium, U: UV light irradiation, B: Blue light irradiation)

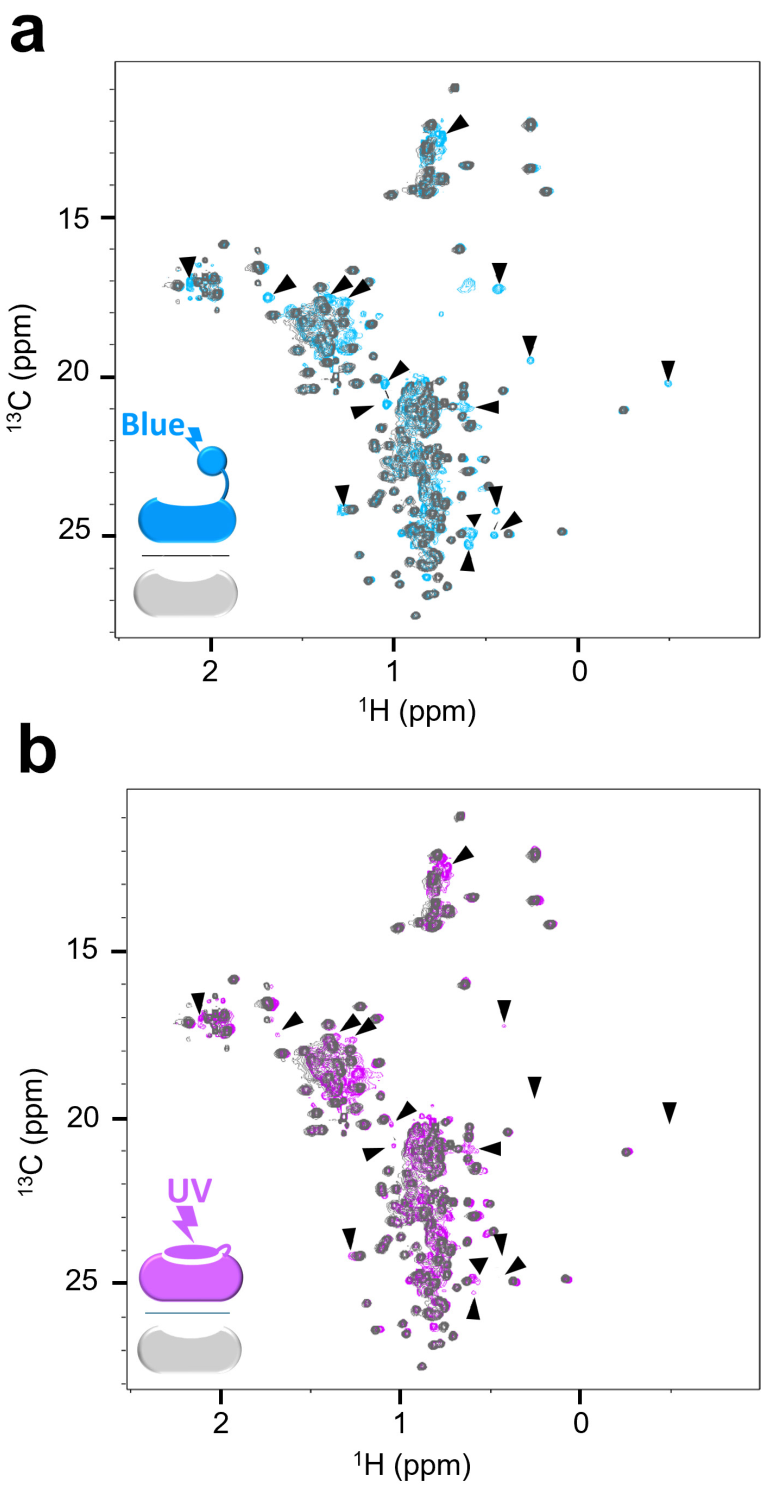

Figure S2. Superposition of ^{1}H–^{13}C methyl HMQC spectra of ON OptoChaperone **(a)** and OFF OptoChaperone **(b)** versus TF$^{PPD\text{-}SBD}$ (gray), respectively. The arrowheads represent GB1-derived signals where significant changes in signal intensity were observed.

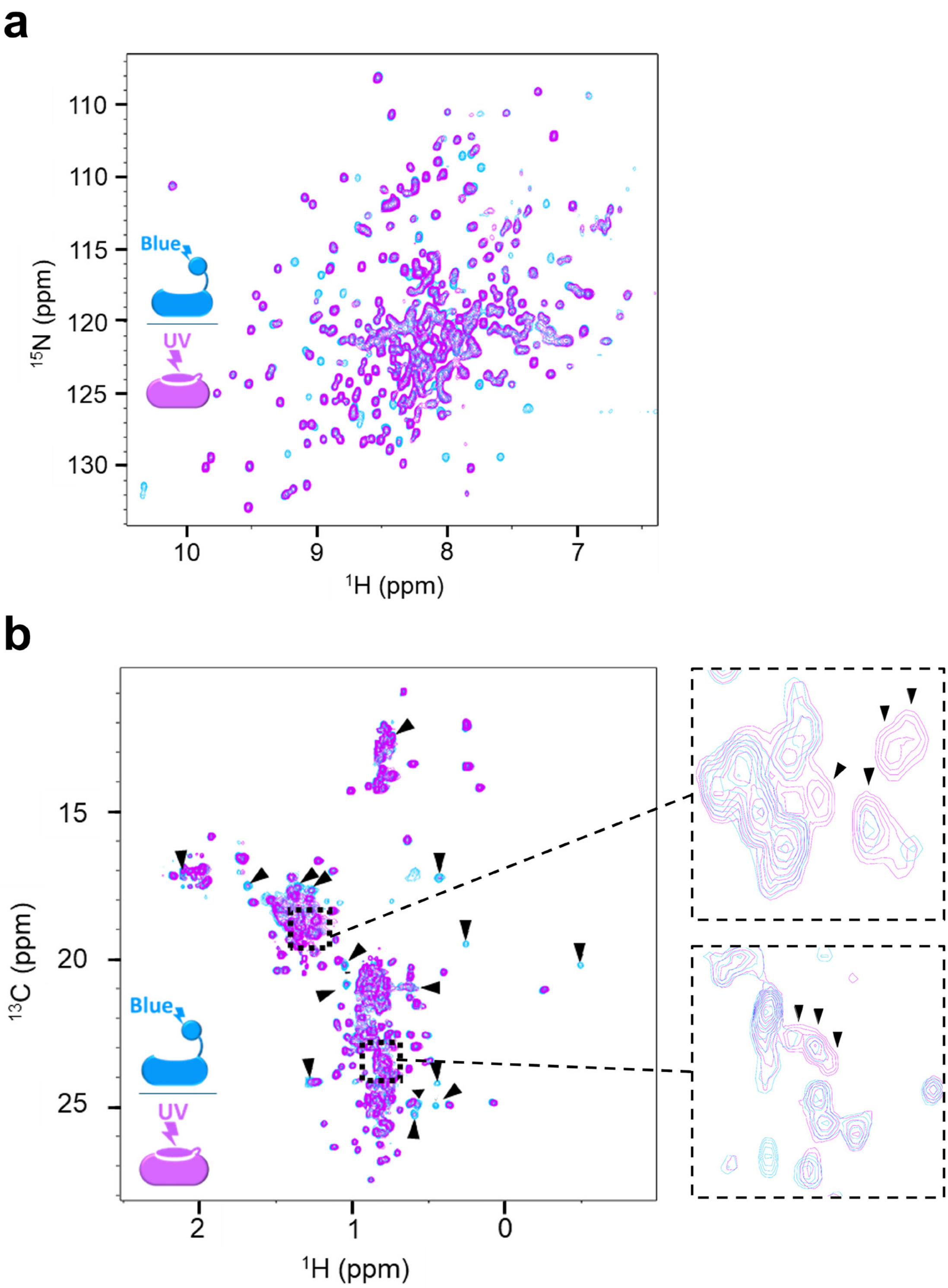

Figure S3. Superposition of the ^{1}H-^{15}N-TROSY-HSQC **(a)** and the ^{1}H-^{13}C-HMQC **(b)** spectra of OptoChaperone after irradiation of blue light (colored blue) or UV light (colored violet). In panel **b**, expanded views of the regions indicated by dotted boxes are provided for clarity.

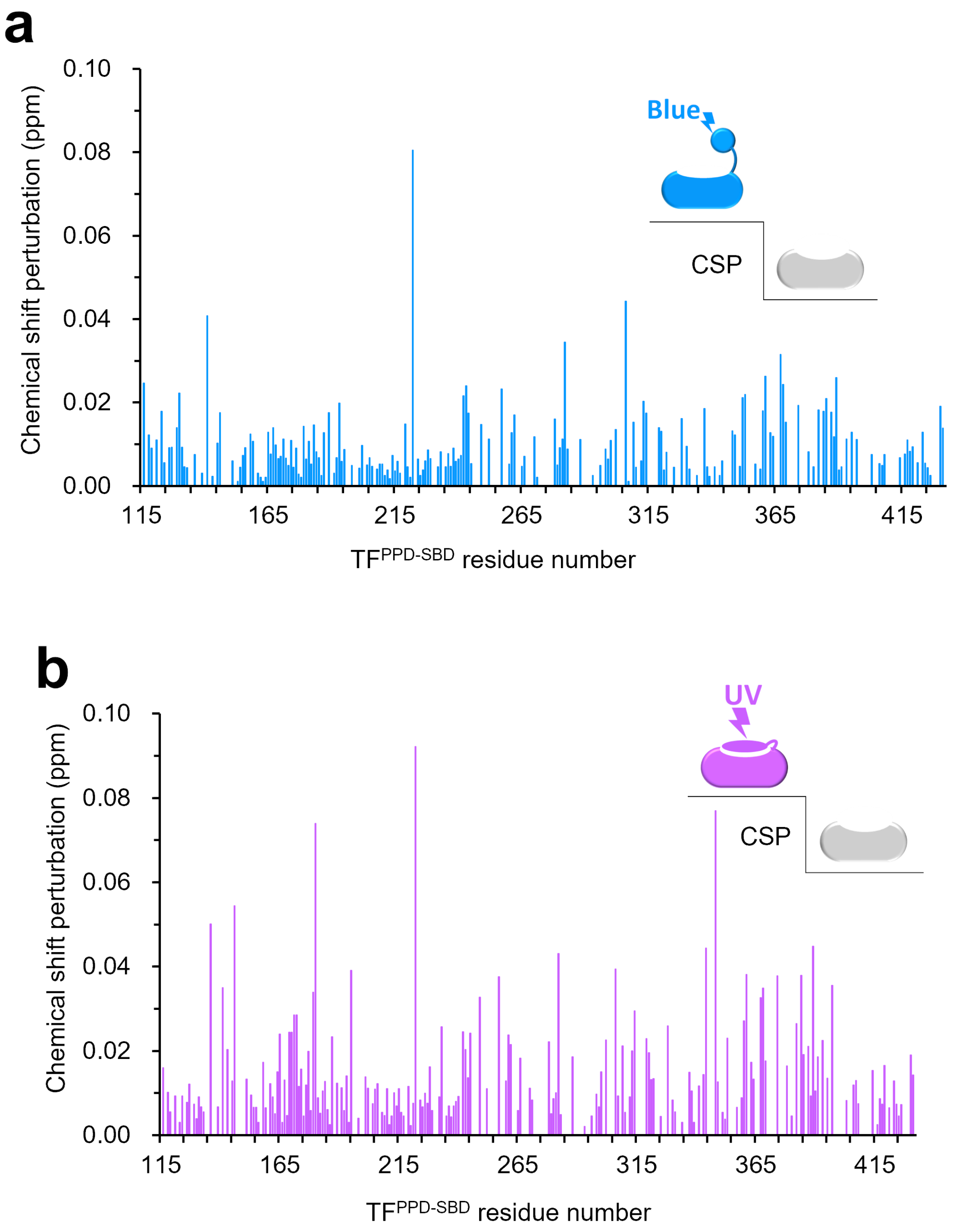

Figure S4. Chemical shift perturbation of amide moieties between **(a)** *trans*-azGB1- TF$^{PPD\text{-}SBD}$ and TF$^{PPD\text{-}SBD}$; **(b)** *cis*-azGB1- TF$^{PPD\text{-}SBD}$ and TF$^{PPD\text{-}SBD}$.

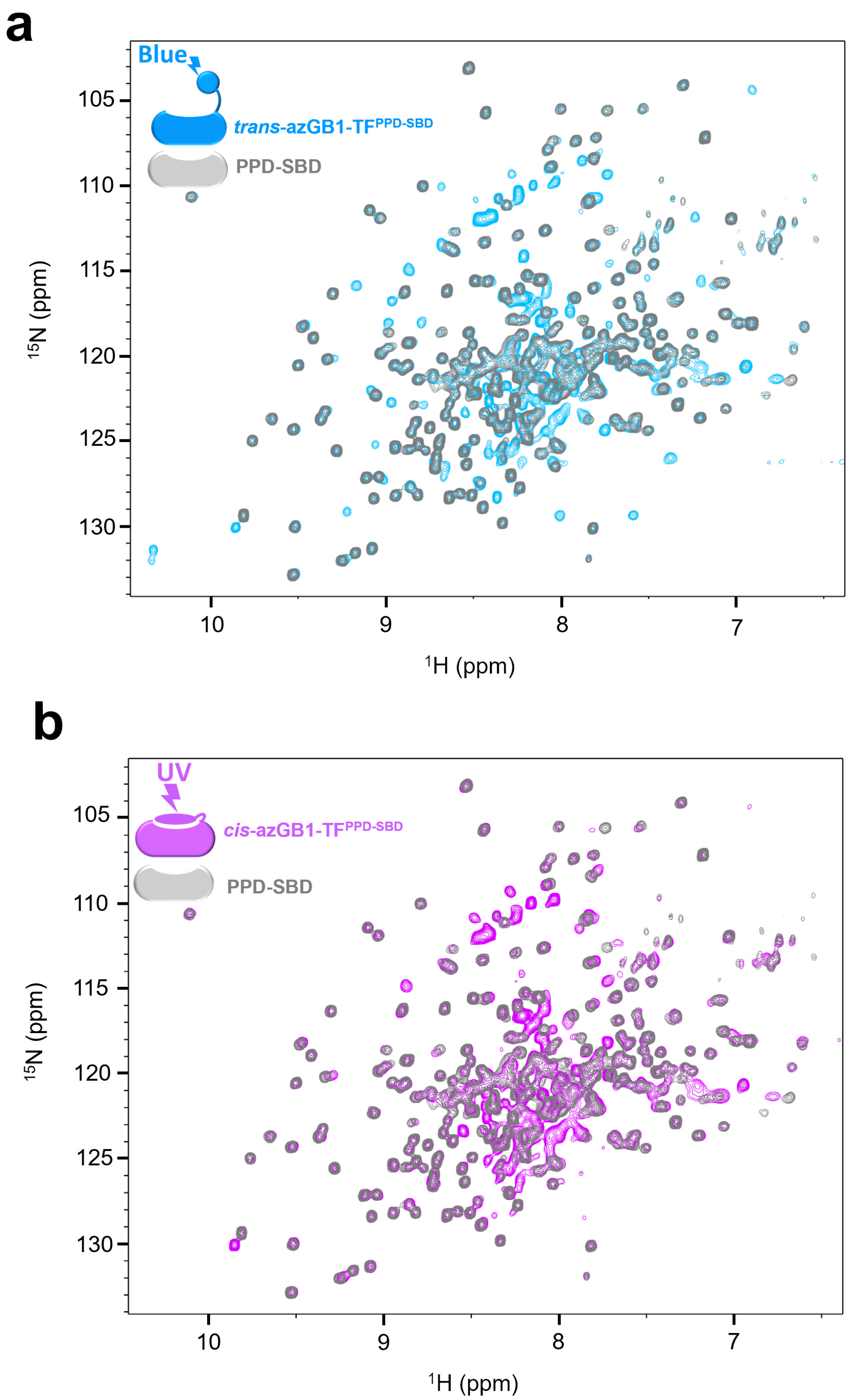

Figure S5. (a) The full ^{1}H-^{15}N-TROSY-HSQC spectra used for the CSD analyses in **Figure S4a**. **(b)** The full ^{1}H-^{15}N-TROSY-HSQC spectra used for the CSD analyses in **Figure S4b**.

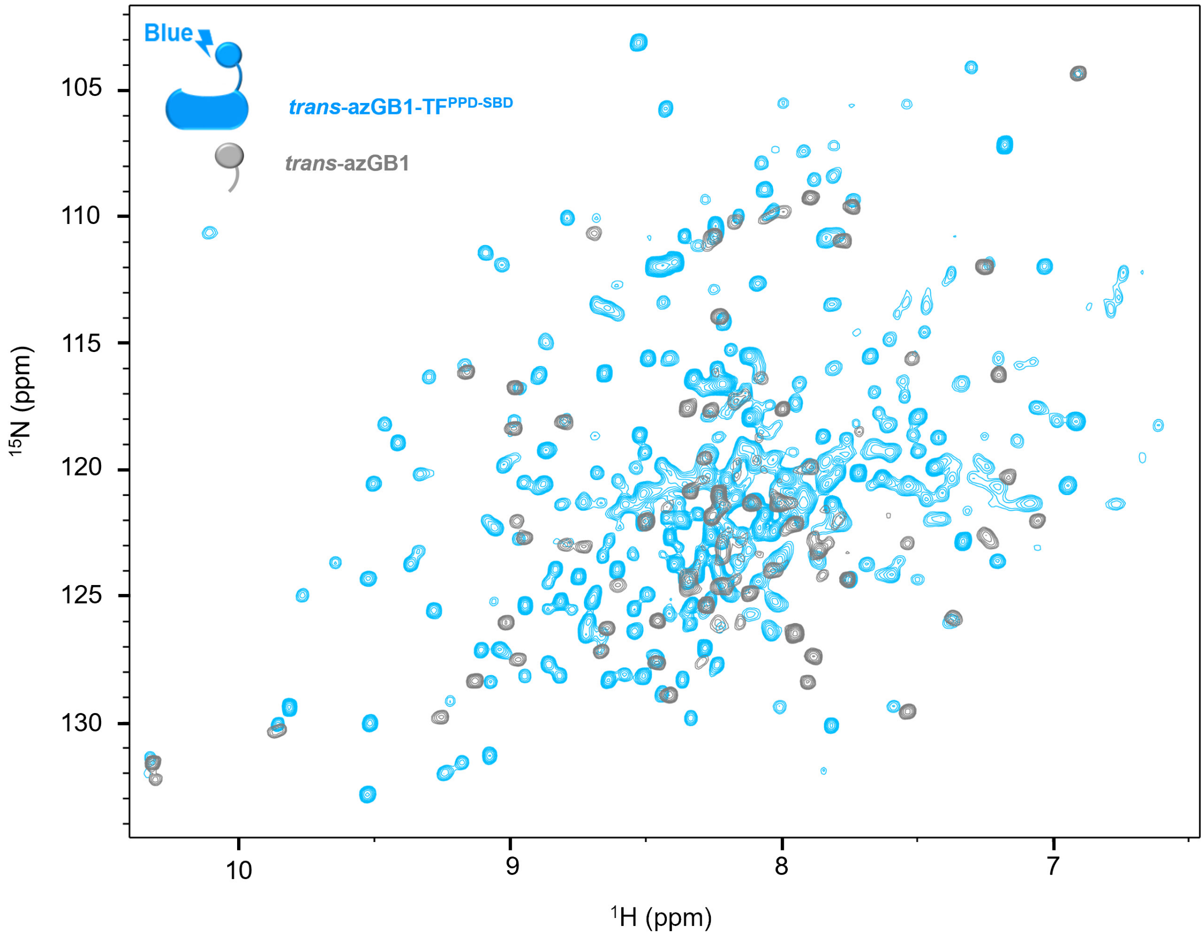

Figure S6. Superposition of ^{1}H-^{15}N-TROSY-HSQC spectra of ^{1}H^{15}N *trans*-azGB1 (gray) with ^{1}H-^{15}N-TROSY-HSQC spectra of ^{2}H^{15}N *trans*-OptoChaperone (blue).

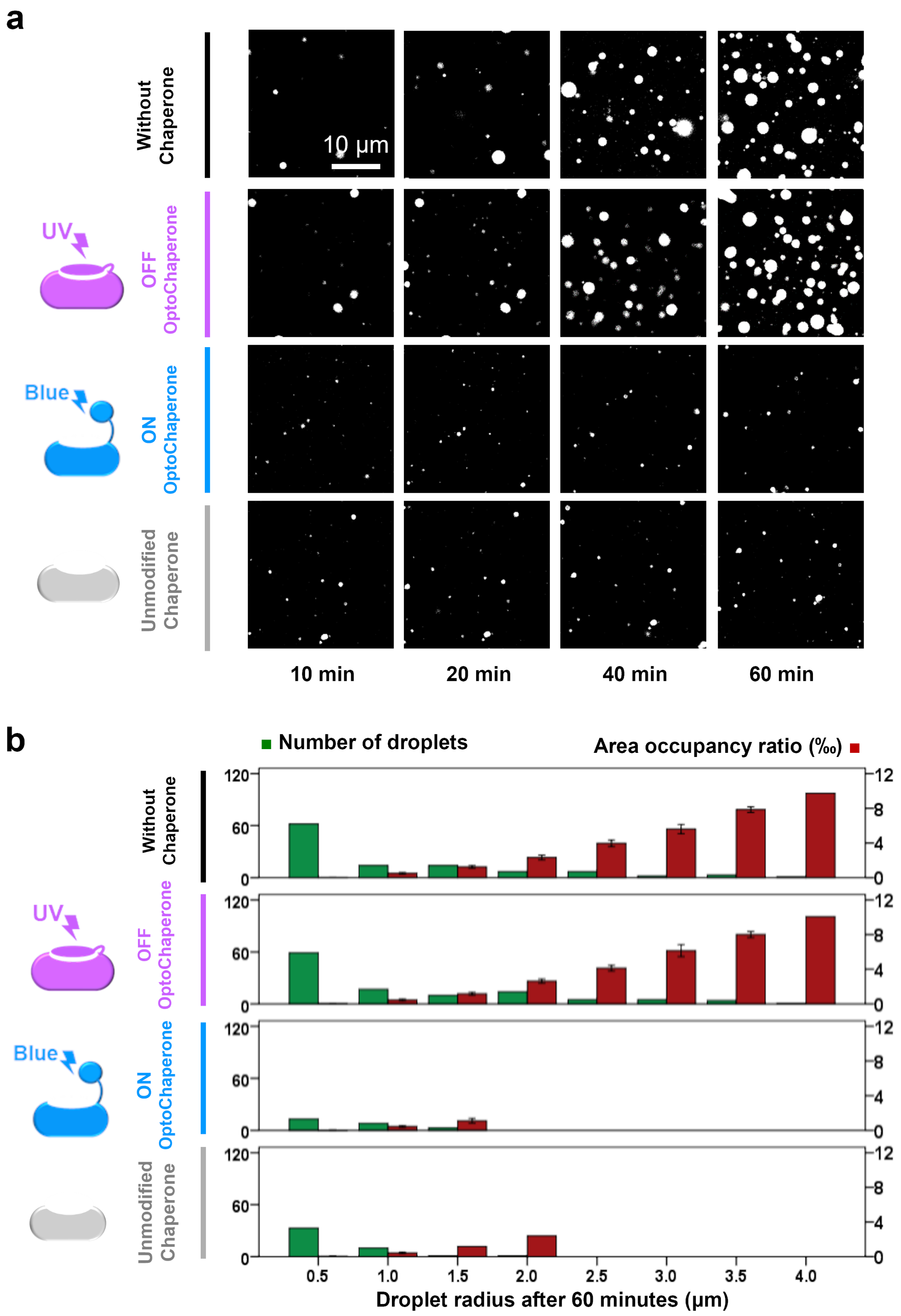

Figure S7. Control of HSF1 condensates in microscopic observation by OptoChaperone. **(a)** Confocal microscopy of HSF1 droplets formation. In the OFF OptoChaperone and ON OptoChaperone conditions, 5-minute UV (OFF) or Blue (ON) light irradiation was

performed. Samples contain HSF1 proteins (20 μM with 10% GFP-labeled HSF1) with or with equivalent concentrations of OFF OptoChaperone, ON OptoChaperone or unmodified chaperone. Droplet formation was triggered by using crowding agents 10% w/v Ficoll. Scale bar: 10 μm. **(b)** Droplet radius (μm) and area occupancy ratio (°/₀₀) at 60 min observed through confocal microscopy. Where area occupancy ratio is the proportion of area that is occupied by each droplet within a field of view on microscope. Bars represent the mean ± s.d. of three independent experiments.

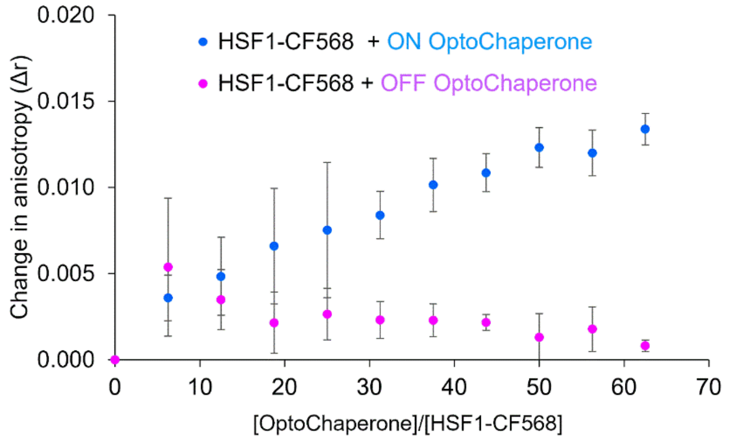

Figure S8. Fluorescence anisotropy analysis of the binding between OptoChaperone and HSF1. HSF1 labeled with CF568 maleimide (1 μM) was incubated in buffer (25 mM HEPES, 150 mM KCl, pH 7.2) with varying concentrations of OptoChaperone that had been pre-irradiated with either blue light (ON state, blue symbols) or UV light (OFF state, red symbols) for 5 min. Measurements were performed at 25 °C (λ_{ex} = 562 nm and λ_{em} = 583 nm). Data are shown as mean ± s.d. (N=3) of $\Delta r = r_{obs} - r_{free}$, where r_{free} is the anisotropy of free HSF1-CF568 and r_{obs} is the anisotropy of HSF1-CF568 measured in the presence of ON or OFF OptoChaperone.

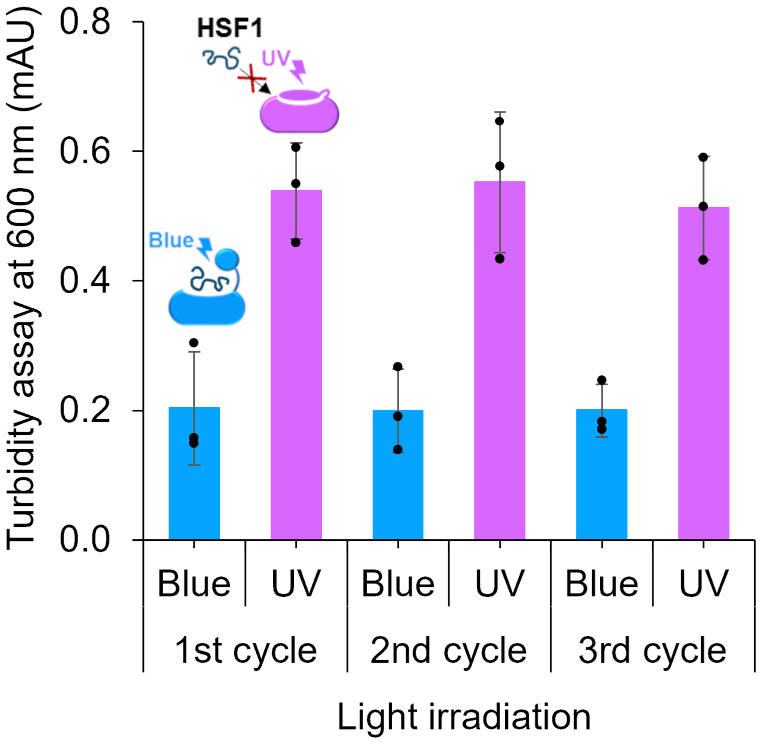

Figure S9. Control of HSF1 condensates in turbidity assay by OptoChaperone. From time-course turbidity measurements in **Figure 3b**, turbidity assay of HSF1 solution at 600 nm at the 30-min time point after 5 min irradiation to UV and blue light. Samples contain HSF1 proteins (20 μM) with equivalent concentrations of OptoChaperone (OFF, ON). Droplet formation was triggered by using crowding agents 10% w/v Ficoll. Bars represent the mean ± s.d. of three independent experiments.

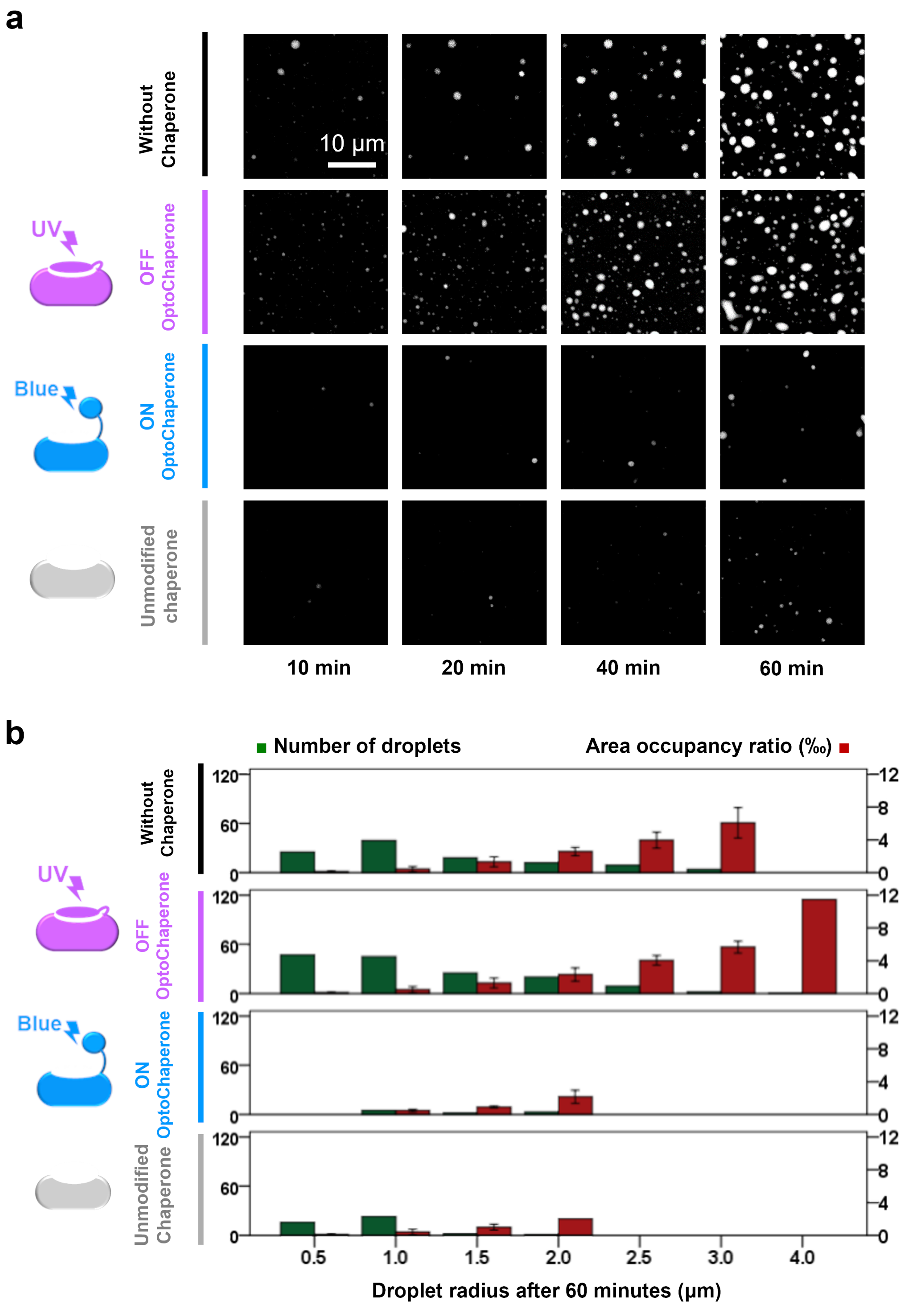

Figure S10. Control of FUS condensates in microscopic observation by OptoChaperone. **(a)** Confocal microscopy of FUS droplets formation. In the OFF OptoChaperone and ON OptoChaperone conditions, 5-min UV (OFF) or Blue (ON) light irradiation was performed.

Samples contain FUS proteins (50 μM with 10% CF488A-maleimide-labeled FUS) with or with equivalent concentrations of OFF OptoChaperone, ON OptoChaperone or unmodified Chaperone. Droplet formation was triggered by using crowding agents 4% w/v PEG8000. Scale bar: 10 μm. **(b)** Droplet radius (μm) and area occupancy ratio (‰) at 60 min observed through confocal microscopy. Where area occupancy ratio is the proportion of area that is occupied by each droplet within a field of view on microscope. Bars represent the mean ± s.d. of three independent experiments.

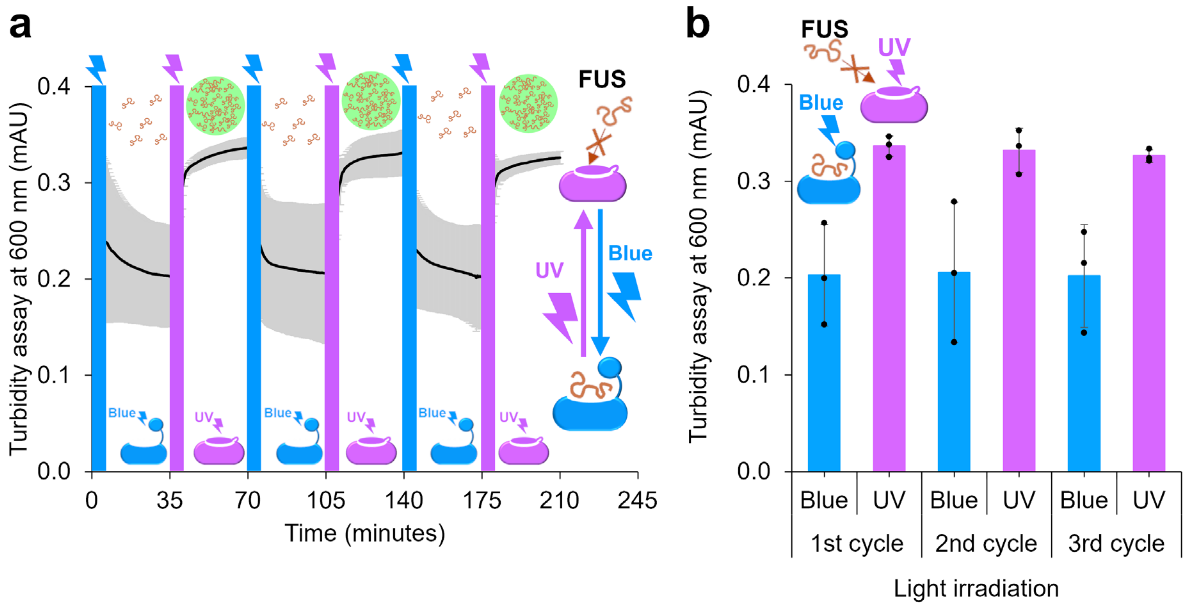

Figure S11. Control of FUS condensates in turbidity assay by OptoChaperone. **(a)** Time-course turbidity measurements (600 nm) of FUS solutions during three cycles of alternating UV and blue light exposure (5 min each). Measurements were recorded every 30 s. After 30 min of crowding agent addition, the light irradiation was performed for 5 min, and the turbidity was measured again. The sample solution contains an equal amount of OptoChaperone and FUS protein, 10 mM DTT and 4% w/v PEG8000. Data are presented as mean turbidity ± s.d. from three independent experiments. **(b)** Turbidity assay of FUS solution at 600 nm at the 30-minute time point after 5 min irradiation to UV and blue light. The schematic model of FUS is colored orange. Bars represent the mean ± s.d. of three independent experiments.

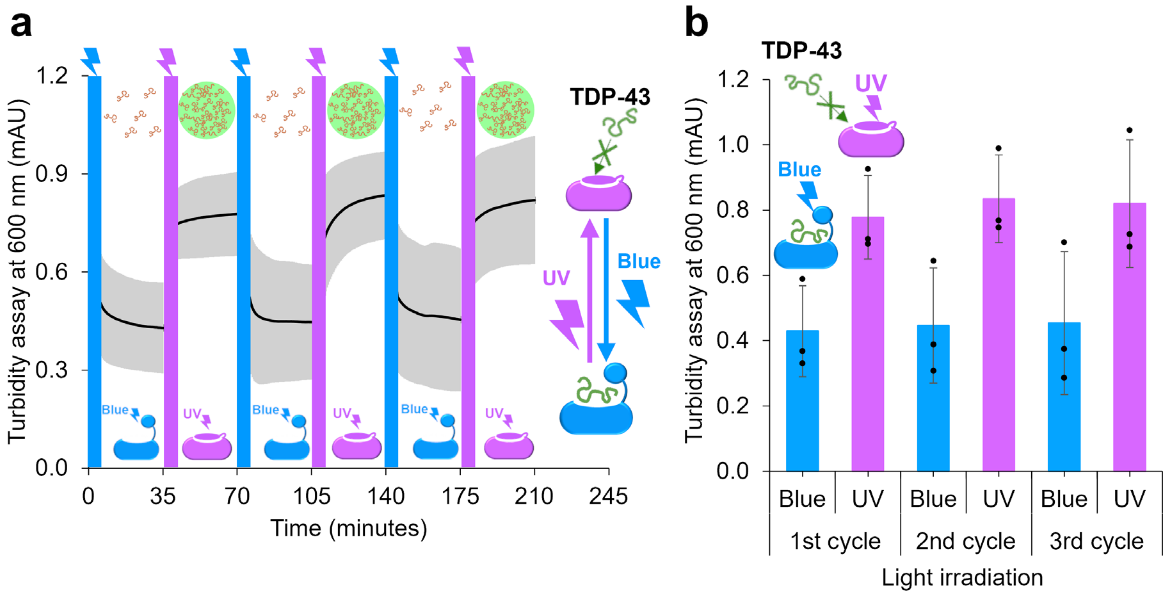

Figure S12. Control of TDP-43 condensates in turbidity assay by OptoChaperone. **(a)** Time-course turbidity measurements (600 nm) of FUS solutions during three cycles of alternating UV and blue light exposure (5 min each). Measurements were recorded every 30 seconds. After 30 min of crowding agent addition, the light irradiation was performed for 5 min, and the turbidity was measured again. The sample solution contains an equal amount of OptoChaperone and TDP-43 protein, 10 mM DTT and 8% w/v PEG8000. Data are presented as mean turbidity ± s.d. from three independent experiments. **(b)** Turbidity assay of TDP-43 solution at 600 nm at the 30-min time point after 5 min irradiation to UV and blue light. The schematic model of TDP-43 is colored green. Error bars: standard deviation. Bars represent the mean ± s.d. of three independent experiments.

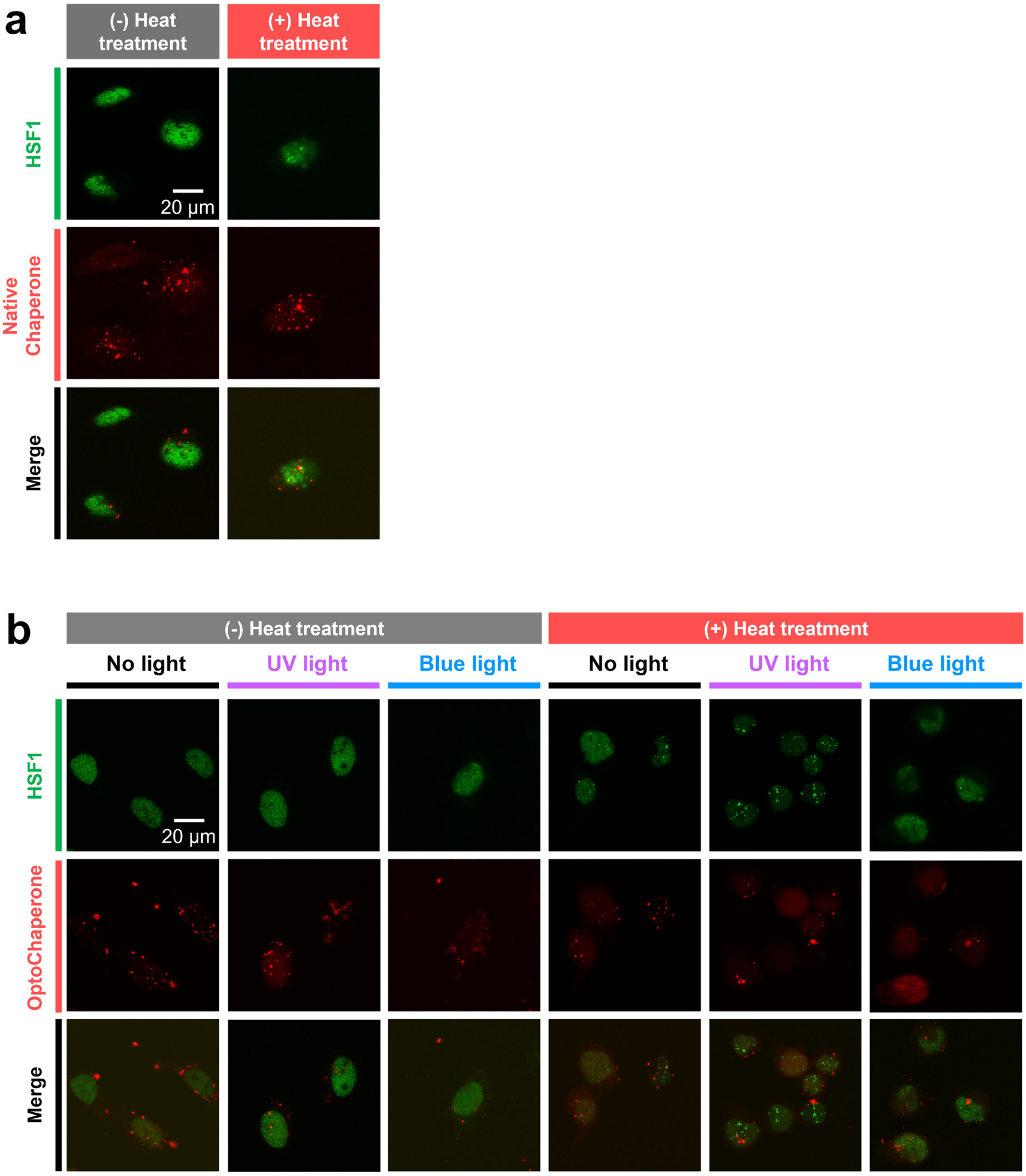

Figure S13. Characterization of OptoChaperone puncta and their spatial relationship with HSF1 foci. **(a)** Confocal microscopy images of HeLa cells electroporated with native chaperone (TF without BSBCA modification). Cells were immunostained for endogenous HSF1 (green). Native chaperone was labeled with ATTO 633 (red). Native chaperone diffused throughout the cells and some portion of them formed cytosolic and nuclear puncta Scale bar: 20 μm. **(b)** Co-localization analysis of OptoChaperone puncta and HSF1 foci under heat stress.

Endogenous HSF1 was visualized as in **(a)**. OptoChaperone was labeled with ATTO 633 (red). OptoChaperone showed a similar distribution and puncta formation to Native TF as in **(a)**, indicating that puncta formation is an intrinsic property of the exogenously introduced TF protein. The merged image demonstrates that OptoChaperone puncta do not colocalize with HSF1 foci, confirming that these puncta are spatially distinct compartments that do not sequester the target HSF1 protein. Scale bar: 20 μm.

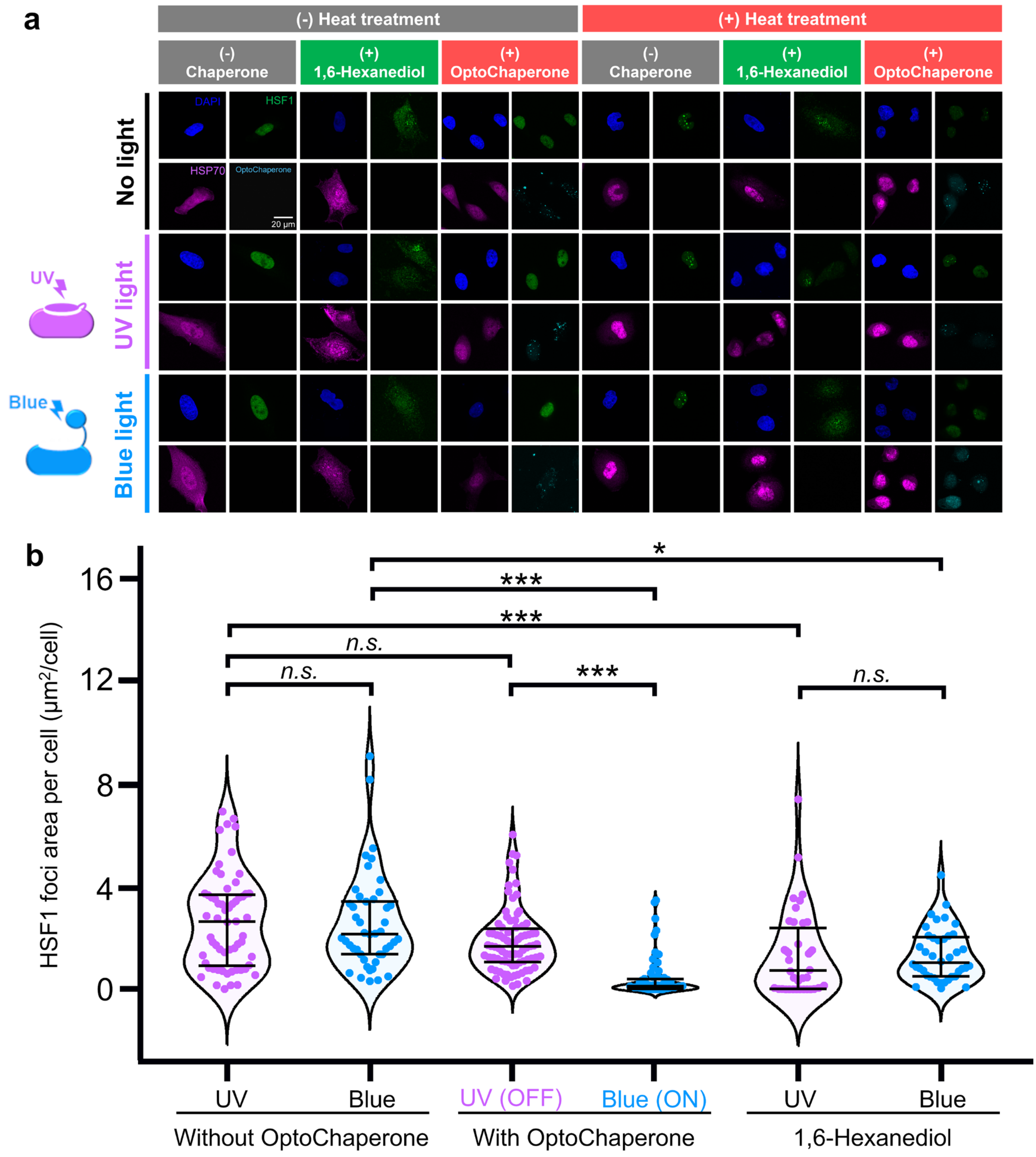

Figure S14. Foci formation of endogenous HSF1 in cells under the heat treatment. **(a)** Confocal microscopy images of HSF1 foci formation under heat stress without, with OptoChaperone, or with 1,6-hexandiol. Endogenous HSF1 was stained using anti-HSF1 antibody (green). The cells were co-stained with DAPI (nucleus, blue) and anti-HSP70 antibody (magenta). OptoChaperone was modified with fluorescent dye (ATTO 633, cyan). Scale bar: 20 μm. **(b)** Quantitative analysis of foci HSF1 formation in cells. The violin plot

displays the distribution of individual data points (small dots) pooled from three independent experiments, overlaid with the median and 25th–75th percentiles (Without OptoChaperone: UV n = 60; Blue n = 45; With OptoChaperone: UV n = 82; Blue n = 62; 1,6-Hexanediol: UV n = 38; Blue n = 40). Statistical significance was determined by the Kruskal-Wallis test followed by Dunn's multiple comparisons test (*n.s.*, not significant; *$p < 0.05$, ***$p < 0.001$).

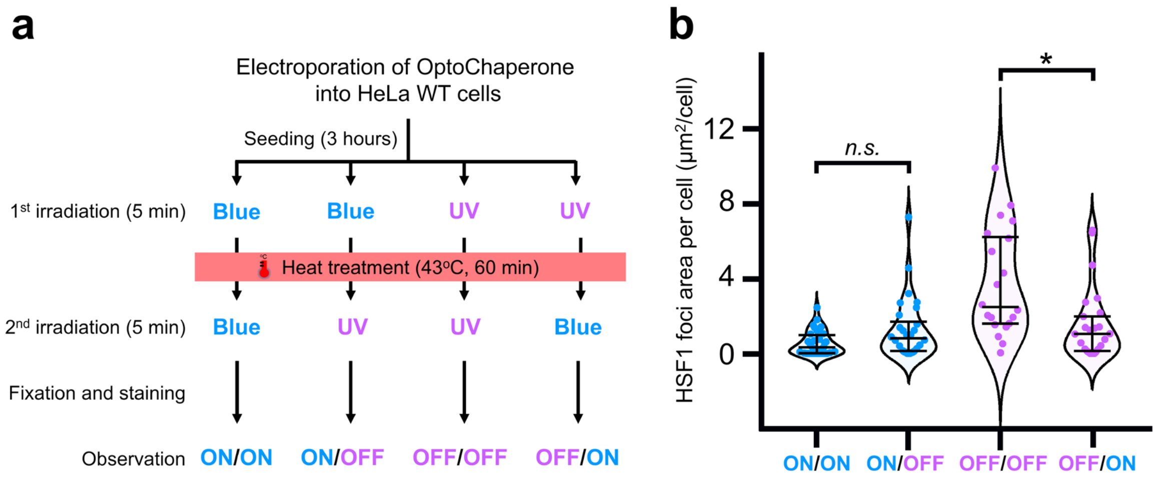

Figure S15. Reversible effect of OptoChaperone on HSF1 foci formation. **(a)** Schematic of the experimental workflow for observing the reversibility of fixed intracellular foci formation. HeLa cells were electroporated with OptoChaperone, incubated for 3 h at 37 °C, and then exposed to either 5 min of UV or blue light. All groups were subsequently subjected to 60-min heat shock, then exposed to either 5 min of UV or blue light again, fixed, immunostained, and observed. **(b)** Quantitative analysis of foci HSF1 formation in cells. The violin plot displays the distribution of individual data points (small dots) pooled from three independent experiments, overlaid with the median and 25th–75th percentiles (Blue – Blue [ON/ON] n = 34; Blue – UV [ON/OFF] n = 28; UV – UV [OFF/OFF] n = 20; UV – Blue [OFF/ON] n = 25). Statistical significance was determined by the Kruskal-Wallis test followed by Dunn's multiple comparisons test (*n.s.*, not significant; *$p < 0.05$).

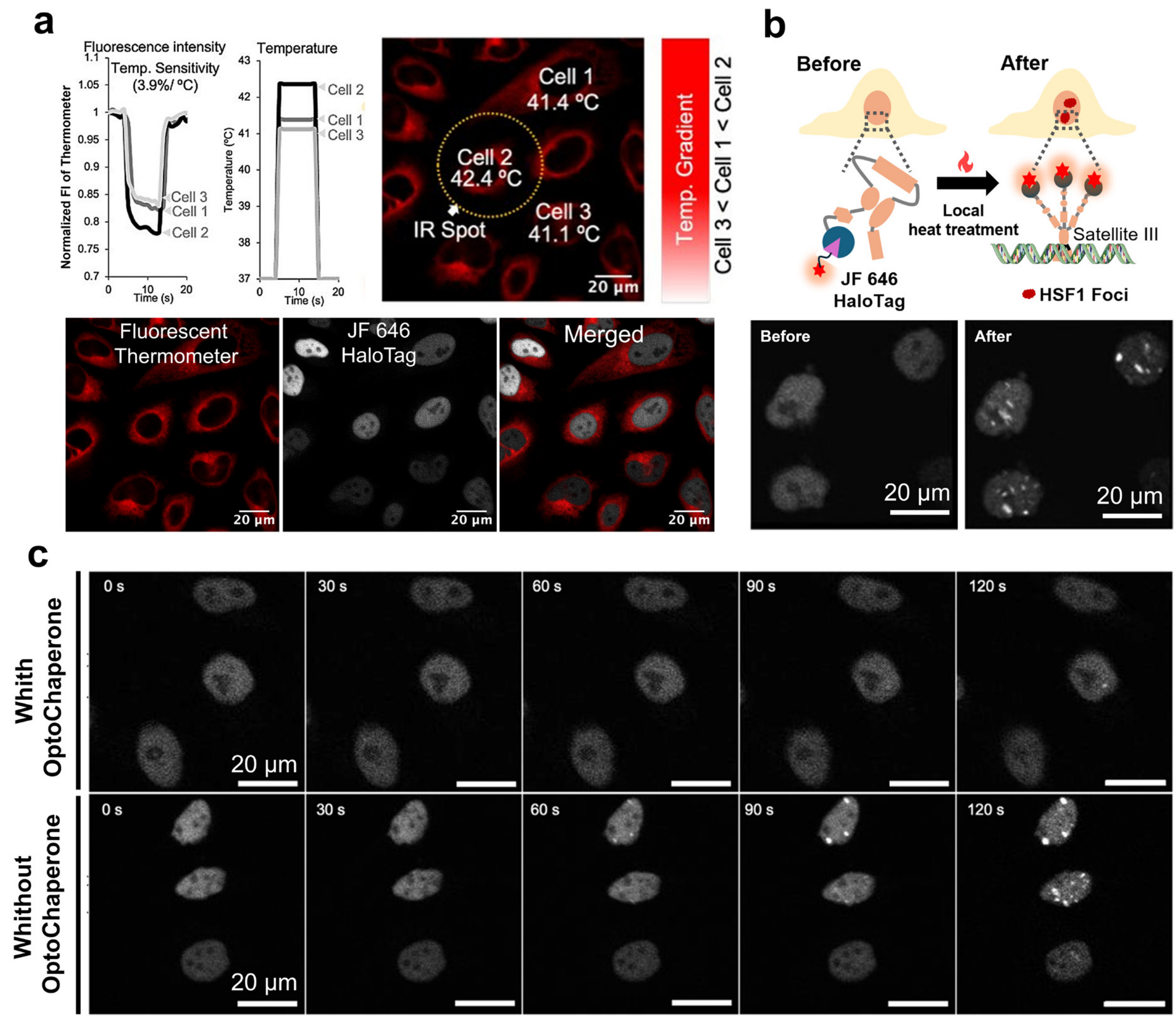

Figure S16. Optochaperone-dependent regulation of heat-induced HSF1 foci formation in living cells. **(a)** Quantification of intracellular temperature. Left panel indicates how the fluorescence intensity change of the fluorescent thermometer (ER Thermo Yellow) was converted to temperature using a sensitivity of 3.9% per °C (upper panel). The right panel illustrates the temperature gradient generated by localized laser heating with a 1470 nm IR laser, where cells positioned within the laser spot (Cell 2) exhibited higher temperatures than cells located outside the heated IR laser spot. Scale bar: 20 μm. **(b)** Schematic depiction of heat-induced HSF1 foci formation at nuclear regions under thermal stress, with representative live-cell fluorescence images shown in the lower panel. Scale bar: 20 μm. **(c)** Real-time observation of HSF1 foci formation in living HeLa cells under heat treatment and

OptoChaperone modulation. HeLa cells expressing HaloTag-fused HSF1 (labeled with 50 nM JF646) were subjected to localized heating at approximately 43°C for 120 s using IR laser. Scale bars: 20 μm. ON OptoChaperone (Top): Cells irradiated with UV/Blue light to activate OptoChaperone show delayed formation of HSF1 foci, OFF OptoChaperone (Bottom): In the absence of OptoChaperone activation, HSF1 foci appear earlier (at 60 s) and exhibit rapid growth in size over the 120-s period.

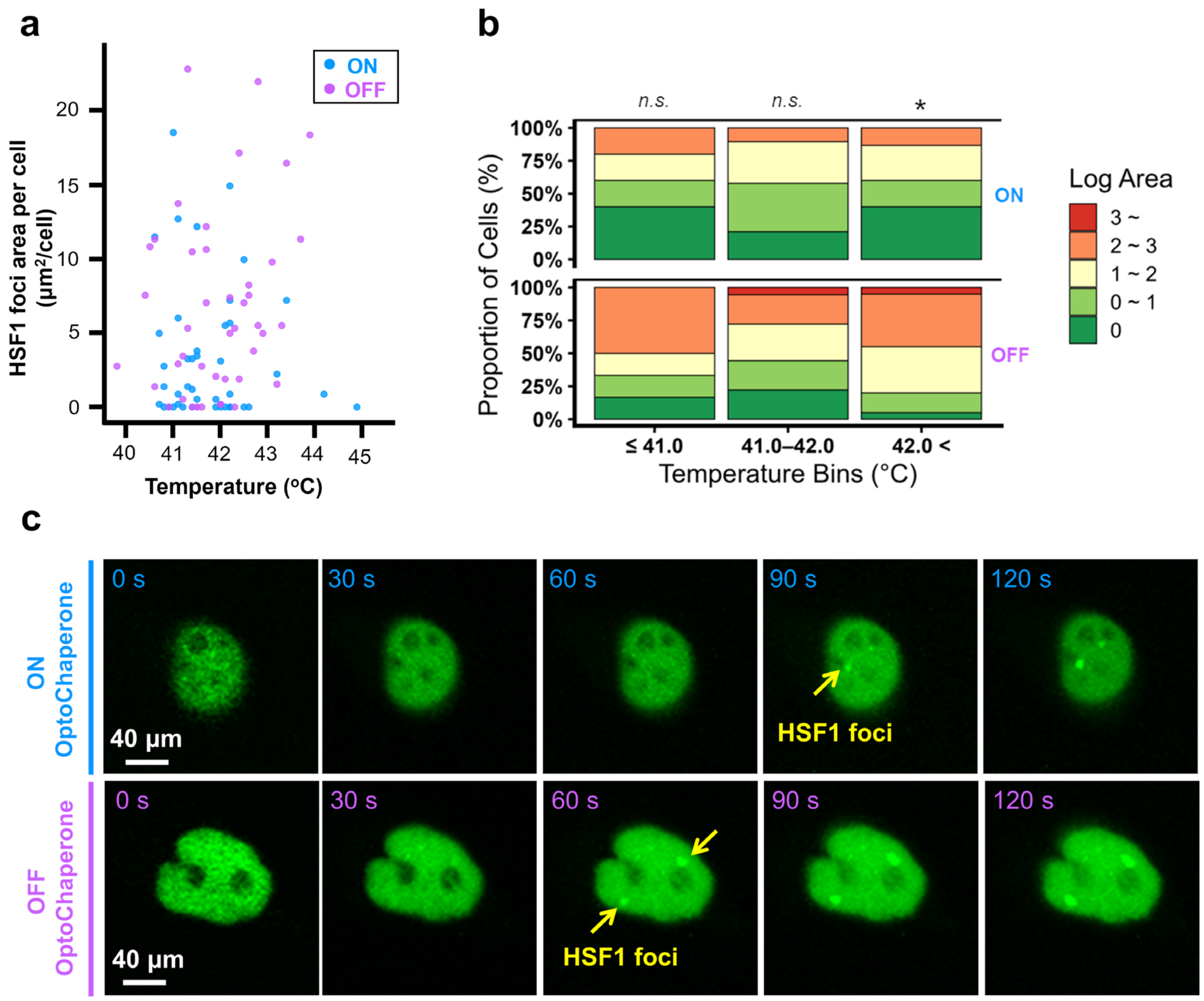

Figure S17. HSF1 foci formation and local temperature at the single-cell level. **(a)** Local temperature and HSF1 foci formation were analyzed using HeLa HSF1-Halo cell lines. Cells were fluorescently labeled with 50 nM JF646 HaloTag ligand for 30 min, followed by loading with ER Thermo Yellow (500 nM) for 30 min. After washing with PBS, HSF1 foci formation was monitored. While temperatures at the single-cell level were determined by fluorescent thermometer, the correlation between intracellular temperature and the area of HSF1 foci were plotted. The scatter plot displays individual data points (small dots) pooled from three independent experiments. **(b)** Stacked bar plot illustrating the proportion of cells in each foci area category across three temperature bins. Area categories are based on the log-transformed values derived from **(a)**. The number of cells in each bin is as follows: ≤ 41.0°C (ON: n = 10, OFF: n = 6), 41.0–42.0°C (ON: n = 19, OFF: n = 18), > 42.0 °C (ON: n = 15,

OFF: n = 20). Statistical significance between the ON and OFF underlying continuous distributions within each bin was determined by the bootstrap Kolmogorov-Smirnov test and adjusted using the Holm-Bonferroni method (*n.s.*, not significant; $^*p < 0.05$). **(c)** Detailed time-lapse observation of HSF1 foci formation at the single-cell level (expanded time-course of **Figure 4e**). HeLa cells expressing HaloTag-fused HSF1 (labeled with 50 nM JF646; green) were subjected to localized heating at 43°C for 120 s using IR laser. This higher-temporal-resolution image sequence demonstrates that the ON OptoChaperone state (Top) delays the formation of HSF1 foci, whereas the OFF state (Bottom) permits the earlier appearance and rapid growth of foci over the 120-s period.

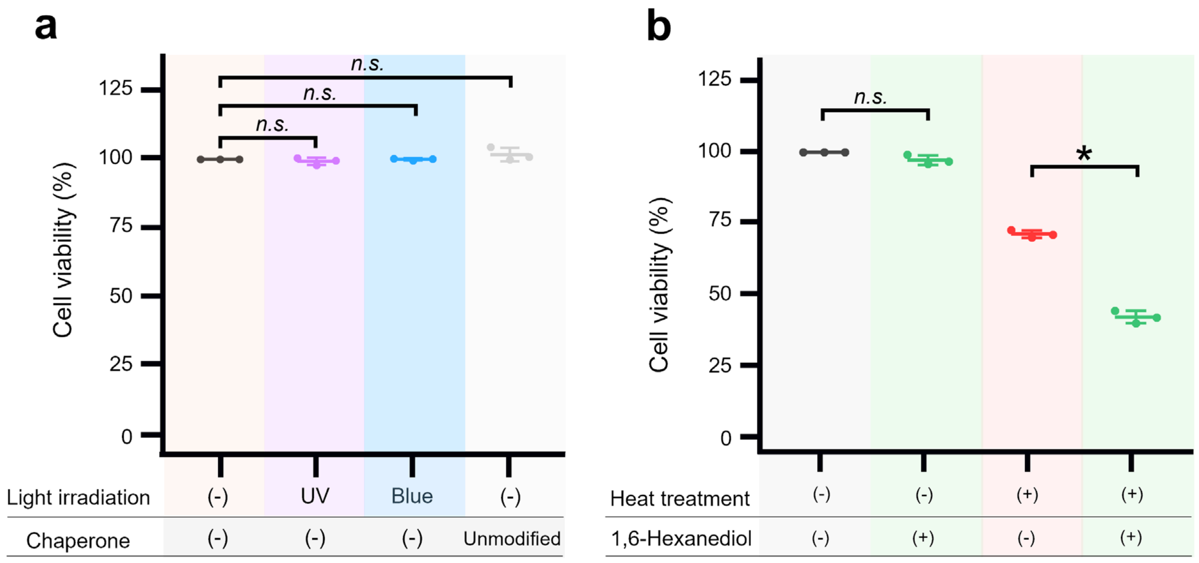

Figure S18. Comparative effects of UV light, blue light and 1,6-Hexanediol on Cell Viability. **(a)** The effect of UV light irradiation, blue light irradiation, or unmodified chaperone introduction on cell viability. Wild-type HeLa cells and HeLa cells electroporated with unmodified chaperone were seeded in a collagen type I coated microplate. Following adherence, some groups of cells were exposed to UV or blue light for 5 min. After subsequent 48 h incubation, cell viability was evaluated by WST-8 assay (absorbance at 450 nm). The plots show individual data points and the mean ± s.d. (N = 3). Statistical significance was determined by one-way ANOVA with Tukey's HSD (*n.s.*, not significant). **(b)** The effect of 1,6-hexanediol on cell viability under conditions with and without heat treatment. Wild-type HeLa cells were seeded in a collagen type I coated microplate. Following adherence, cells were incubated at 37 °C or 43 °C (heat treatment) with or without 0.1% w/v 1,6-hexanediol. After subsequent 48-h incubation, cell viability was evaluated using a WST-8 reagent and measured by absorbance at 450 nm. The plots show each data points, and the mean ± s.d. (N = 3). Statistical significance was determined by one-way ANOVA followed by Tukey's HSD (*n.s.*, not significant; *p < 0.05).

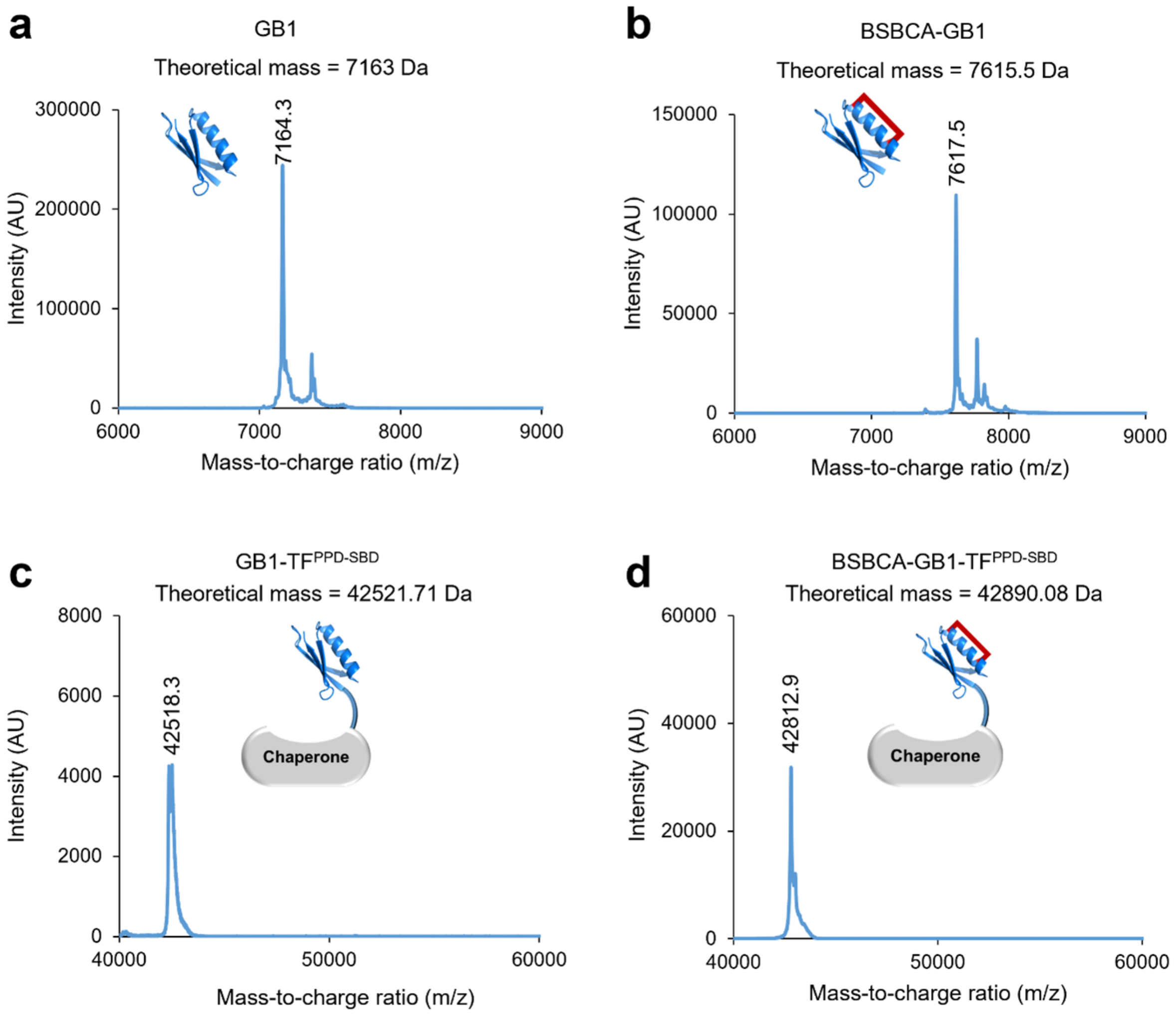

Figure S19. Matrix-Assisted Laser Desorption/Ionization-Time of Flight-Mass Spectrometry (MALDI-TOF-MS) analysis of GB1 and GB1-TF$^{PPD-SBD}$ fusion proteins before and after BSBCA modification. **(a)** Mass spectrum of unmodified GB1 protein measured by MALDI-TOF-MS. **(b)** Mass spectrum of BSBCA-modified GB1 (BSBCA-GB1) protein measured by MALDI-TOF-MS, showing the expected mass increase corresponding to the BSBCA modification. **(c)** Mass spectrum of unmodified GB1-TF$^{PPD-SBD}$ fusion protein measured by MALDI-TOF-MS. **(d)** Mass spectrum of BSBCA-modified GB1-TF$^{PPD-SBD}$ fusion protein (BSBCA-GB1-TF$^{PPD-SBD}$) measured by MALDI-TOF-MS. In all cases, the observed masses correspond well to the theoretical values, confirming both the identity of the purified proteins and the successful BSBCA modification where applicable.

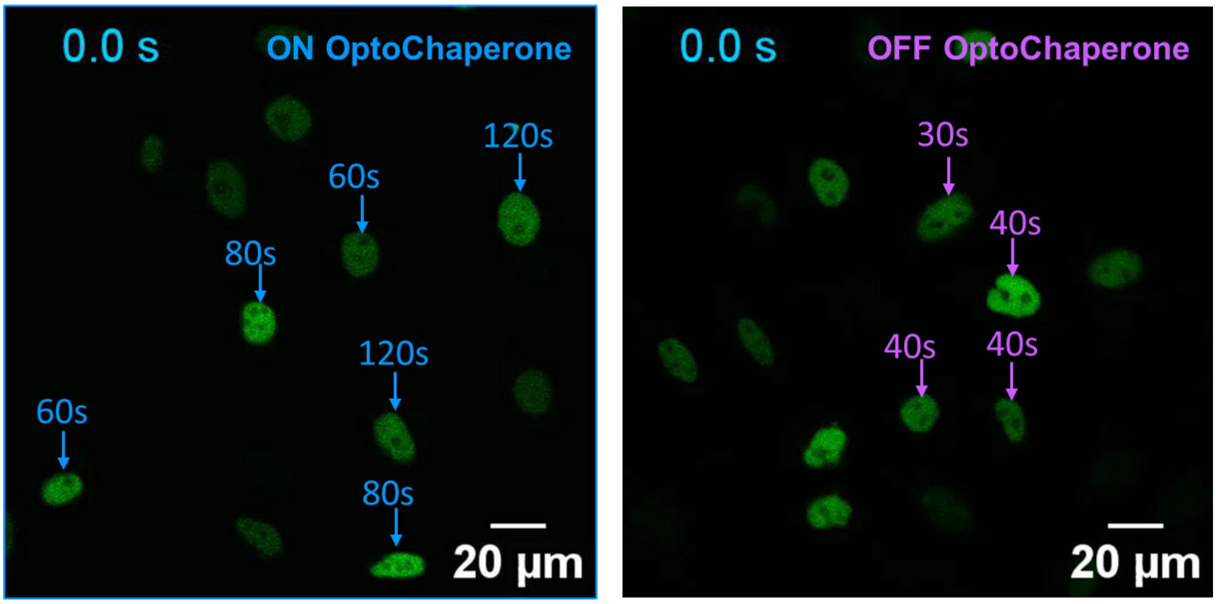

Movie M1. Real-time observation of HSF1 foci formation. Spatiotemporal monitoring of HSF1 foci formation in living HeLa cells modulated by OptoChaperone. HeLa cells expressing HaloTag-fused HSF1 (labeled with 50 nM JF646) were subjected to localized heating at 43°C for 120 s using a 1470 nm laser. The video monitors the time to foci formation following the onset of heat treatment under blue light (ON OptoChaperone) and UV light (OFF OptoChaperone) conditions.
